# Disconnection of the late Pliocene Agulhas Leakage from Atlantic Meridional Overturning Circulation

**DOI:** 10.1038/s41561-026-02055-5

**Published:** 2026-08-03

**Authors:** Suning Hou, Alejandra Cartagena-Sierra, Ning Tan, Carolien M. H. van der Weijst, Malte Stockhausen, Fenghao Liu, Melissa A. Berke, Isla S. Castañeda, Aidan Starr, Francien Peterse, Francesca Sangiorgi, Anna S. von der Heydt, Appy Sluijs, Peter K. Bijl

**Affiliations:** 1 Department of Earth Sciences, Utrecht University, Utrecht, the Netherlands; 2 Department of Civil and Environmental Engineering and Earth Sciences, University of Notre Dame, Notre Dame, IN USA; 3 State Key Laboratory of Lithospheric and Environmental Coevolution, Institute of Geology and Geophysics, Chinese Academy of Sciences, Beijing, China; 4 State Key Laboratory of Marine Geology, Tongji University, Shanghai, China; 5 Department of Earth, Geographic and Climate Sciences, University of Massachusetts Amherst, Amherst, MA USA; 6 Department of Geography, University of Cambridge, Cambridge, UK; 7 Institute for Marine and Atmospheric Research Utrecht, Utrecht University, Utrecht, the Netherlands; 8 Present Address: Inigo Insurance, Inigo Ltd, London, UK

**Keywords:** Palaeoceanography, Palaeoclimate, Physical oceanography

## Abstract

The strength of the Atlantic Meridional Overturning Circulation (AMOC), in terms of streamfunction, is considered to depend partly on salt supply from the Indian Ocean through Agulhas Leakage (AL). In turn, AL is regulated by the position of the Southern Ocean subtropical front, which creates the pathway of the leaking around the African continent. Here, to evaluate the links between the subtropical front, AL and AMOC, we present latitudinal shifts of the subtropical front, and thereby potential AL changes, using dinoflagellate cysts and biomarkers in sediment cores from the Agulhas Plateau covering the late Pliocene (3,600–2,580 thousand years ago (ka)). We subsequently compare our frontal migration and AL reconstruction with ocean surface and bottom records along the AMOC pathway, and climate model simulations, to evaluate the influence of the AL on the AMOC. We find that the northward migration of the subtropical front gradually reduced the volume and salt supply of AL from 3,600 ka to 3,300 ka, whereas the AMOC intensified. We conclude that the causal link between the AL and AMOC was disconnected during the late Pliocene, challenging the presumed role of the AL in modulating the AMOC.

## Abstract (web summary)

Northward movement of the Southern Ocean subtropical front diminished salt transport by Agulhas Leakage in the late Pliocene, although this had no apparent influence on the strength of the Atlantic Meridional Overturning Circulation, according to proxy records from marine sediment cores.

## Main

The Agulhas Leakage (AL), consisting of large mesoscale eddies of saline, warm Indian Ocean waters that enter the Atlantic Ocean during the retroflection of the Agulhas Current south of Africa, is thought to be a key component of the Atlantic Meridional Overturning Circulation (AMOC), based on numerical model experiments and observations^1–4^. In the prevailing paradigm, AL is primarily driven by wind forcing: intensified Southern Hemisphere westerlies promote a southward shift of the Antarctic Circumpolar Current (ACC)^5^, displacing the subtropical front (STF) poleward and facilitating increased leakage of Indian Ocean water into the Atlantic^3,4,6^. Thus, STF migration is commonly interpreted as an integrated response to wind stress that regulates AL intensity^7,8^, albeit with spatiotemporal complexities^9,10^.

According to this paradigm, salt supplied to the Atlantic via AL is ultimately transported northwards by surface currents, notably the North Atlantic Current (NAC). The AL is thus critical for establishing the density required for North Atlantic Deep Water (NADW) formation^3,8,11,12^. However, contrasting views exist regarding the role of AL in AMOC under the present-day warming background^2,4,7,13,14^ and uncertainties remain concerning the proportion of AL that ultimately enters the North Atlantic^15^. Furthermore, debate exists over the fate of AMOC itself^16^. Palaeoceanographic data of past climates therefore provide a critical test of the theoretical causal link between AL and AMOC.

The late Pliocene (3,600–2,580 thousand years ago (ka)) is characterized by periods of global warmth, including the mid-Piacenzian Warm Period (mPWP; ∼3,264–3,025 ka)^17^, which is considered to be the most recent geological analogue for a warmer modern world^17–19^. Marine Isotope Stage (MIS) M2 (~3,300 ka), before the mPWP, was a transient global cooling phase seen as a failed attempt for a large-scale Northern Hemisphere glaciation^20,21^ and provides insights into cold-to-warm climate transitions. Most coupled general circulation model simulations focus only on the mPWP (explicitly KM5c at 3,205 ka) and have suggested an AMOC similar to^22^ or stronger than the present AMOC^23,24^. However, mPWP simulation outputs are highly dependent on palaeogeographic boundary conditions, which remain debated^25,26^, and on spatial resolution. Moreover, while extensive research has focused on the upper (NAC)^27–33^ and lower (NADW)^28,34^ limbs of the AMOC, these components are rarely examined in conjunction, and the presumed interhemispheric connectivity through AL remains underexplored. Reconstructions from the Southern Benguela region did not detect clear AL changes^35,36^, underscoring the complexity of the Agulhas system and highlighting the need for improved constraints on AL. Collectively, testing whether variability in AL forced late Pliocene AMOC changes requires reconstructions of the STF and comparison with AMOC reconstructions.

**Fig. 1:**
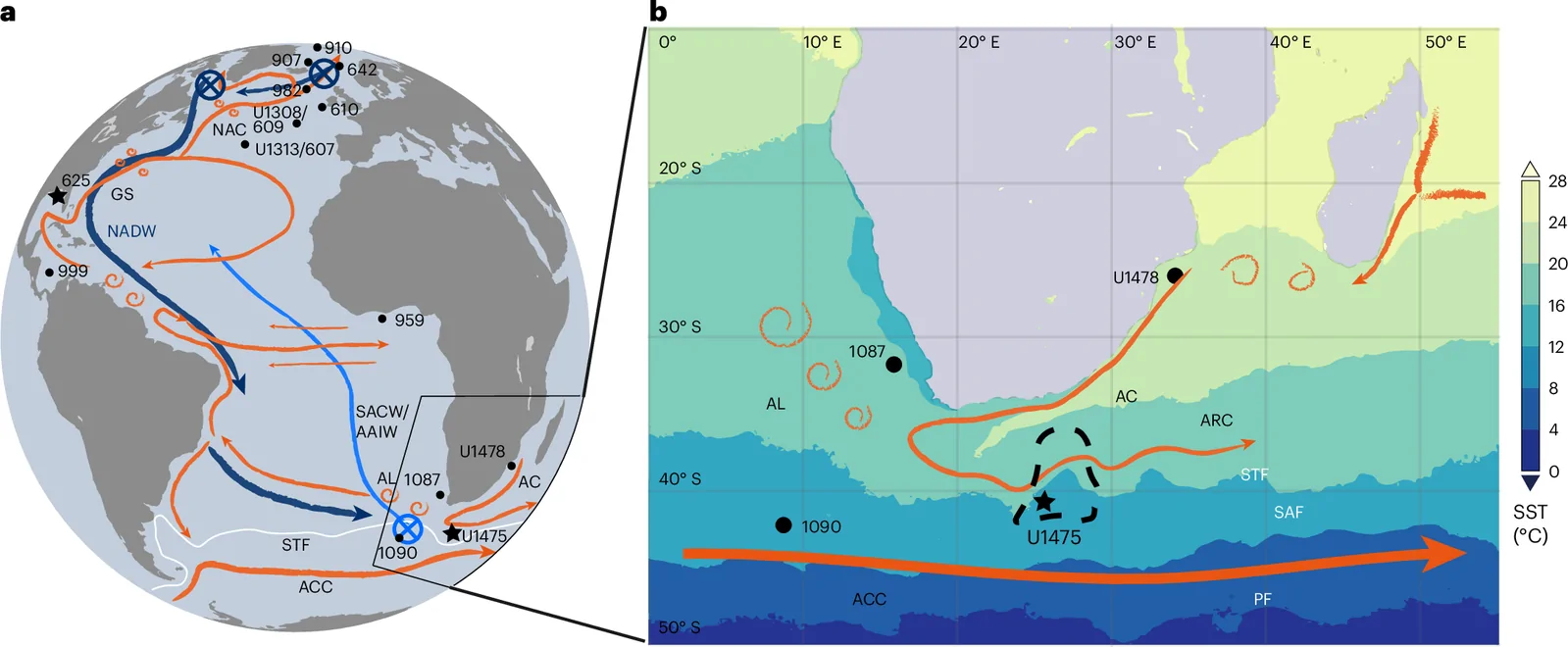
Schematic deep and surface ocean circulation in the Atlantic Ocean and Agulhas. **a**, Conceptual representation of the AMOC and Southern Ocean circulation^72^. The orange arrows indicate surface currents. The blue arrows indicate intermediate and deep ocean water masses. Crosses indicate locations of deep and intermediate water formation. Dots indicate sites with relevant Pliocene data. Stars indicate sites studied. **b**, Agulhas system overlaying modern annual mean SST^73^. The orange lines indicate the Agulhas-associated currents. Circles denote filaments and eddies. The dashed black line denotes the outline of the Agulhas Plateau. Approximate positions of oceanic fronts are marked by SST contours (12 °C, 8 °C and 4 °C isotherms indicate STF, SAF and PF, respectively). AAIW, Antarctic Intermediate Water; AC, Agulhas Current; GS, Gulf Stream; SAF, subantarctic front; PF, polar front. Basemap in **a** created with tmap^74^. Basemap in **b** generated with Giovanni (https://giovanni.gsfc.nasa.gov/giovanni)^75^.

To fill this critical knowledge gap, we focus on reconstructions of the local oceanography at the Agulhas Plateau, which is located between the Agulhas Retrocurrent (ARC) and the STF and thus can record the STF migration. We first generate records of dinoflagellate cyst (dinocyst) assemblages, which distinctively differ between water masses and frontal systems in the Southern Ocean^37^, and biomarker-based sea surface temperature (SST) records (alkenone unsaturation index U^K^^′^_37_ and tetraether index of 86 carbon atoms TEX_86_) for International Ocean Discovery Program (IODP) site U1475 (Fig. 1). From these, we infer latitudinal migrations of the STF, wherein a southward STF position is interpreted as high AL potential, and vice versa^7,11^. To evaluate the connection of AL with AMOC variations, we also generated U^K′^_37_ and TEX_86_ records for Ocean Drilling Program (ODP) site 625 in the northern Gulf of Mexico (GoM) (Fig. 1), where the NAC originates and the NADW passes by. These records allow us to trace changes in current strength and the salinity of the AMOC loop in the context of previous studies. Finally, we revisit numerical modelling outputs of the mPWP and MIS M2 ocean circulation to assess the consistency with our proxy-based reconstructions.

## STF migration during the late Pliocene

**Fig. 2:**
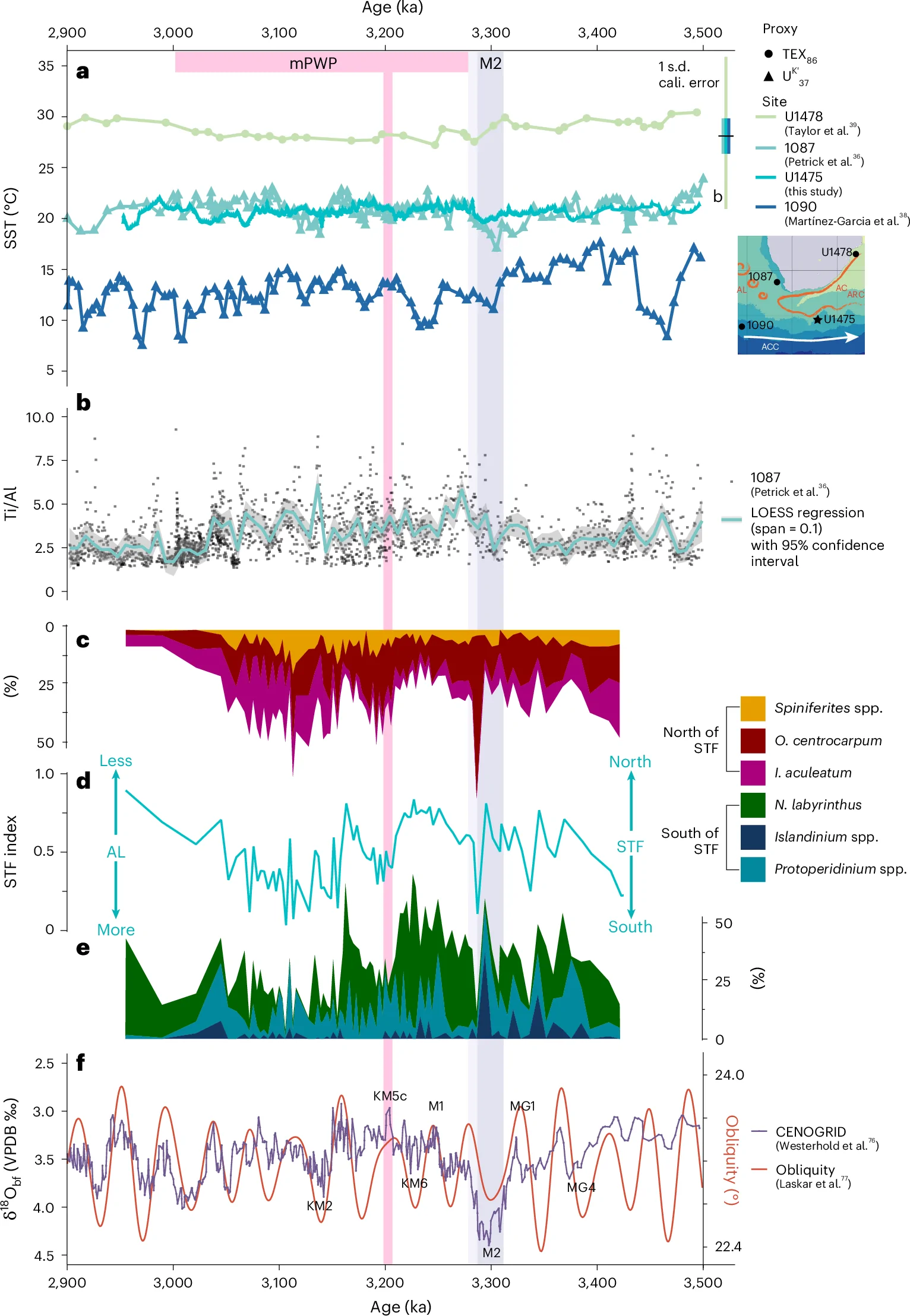
Palaeoceanographic changes at Agulhas Plateau. **a**, SST records for the Agulhas region^36,38,39^ and this study with calibration (cali) error bars indicated. Site U1475 U^K′^_37_ SST is a 4-point moving average as shown in Extended Data Fig. 1. **b**, XRF Ti/Al of ODP site 1087 (ref. ^36^). The teal line denotes LOESS regression (span = 0.1). The grey band denotes the 95% confidence interval. **c**,**e**, Dinocyst assemblages of site U1475 indicating STF migration, primary productivity and sea ice conditions. **d**, STF index (Methods) indicating the relative latitudinal position of the STF and strength of AL. **f**, Global benthic foraminiferal δ^18^O compilation^76^ in Vienna Pee Dee Belemnite with marine isotope stages indicated and obliquity^77^. The pink interval and bar highlight mPWP and MIS KM5c. Darker and lighter purple highlight MIS M2 and its deglaciation stage. Basemap in **a** generated with Giovanni (https://giovanni.gsfc.nasa.gov/giovanni)^75^.

**Fig. 3:**
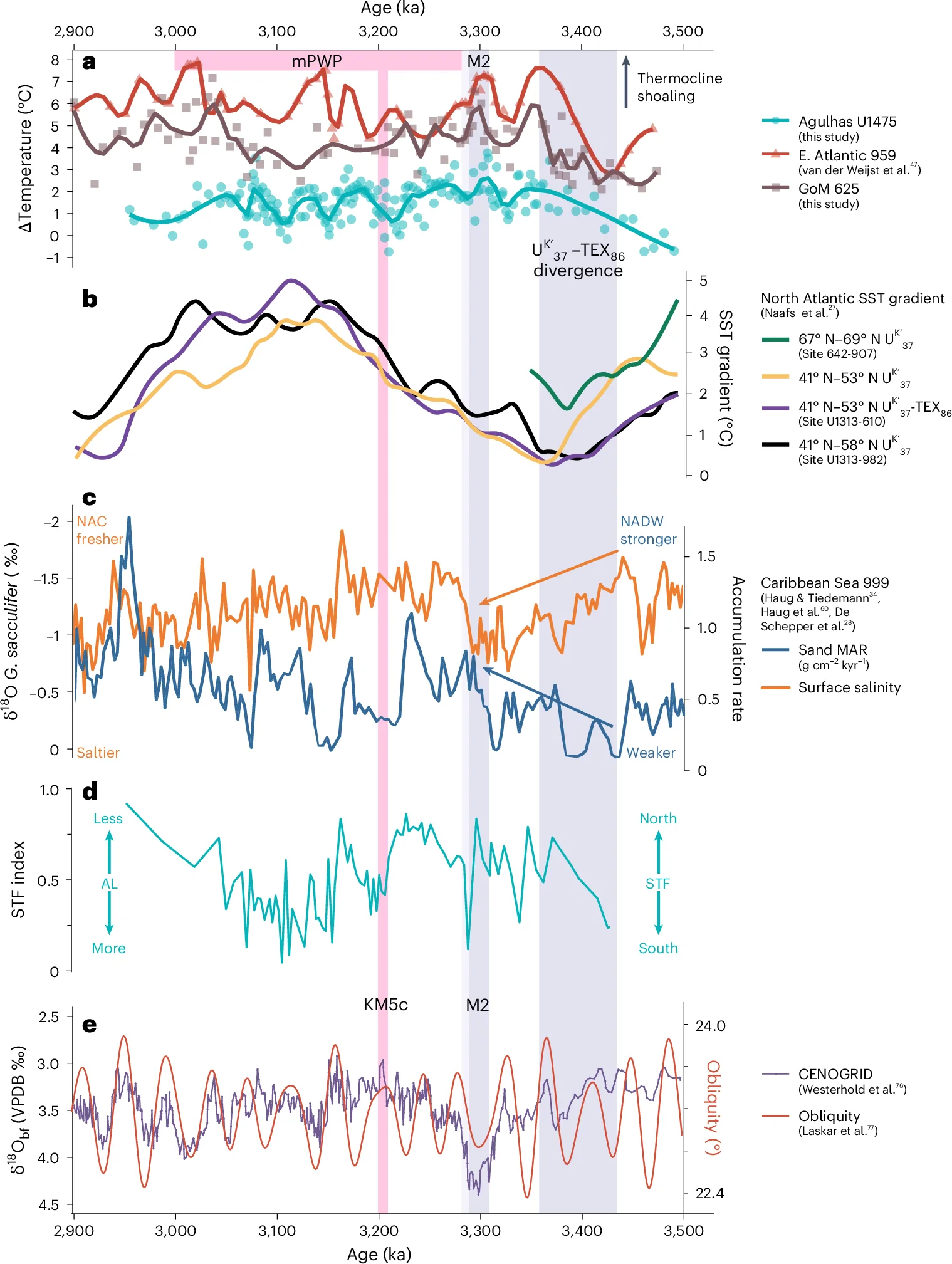
Palaeoceanographic changes associated with the AMOC. **a**, Difference between U^K′^_37_- and TEX_86_-derived SSTs for IODP site U1475 (teal, this study), East Atlantic ODP site 959 (red)^47^ and ODP site 625 (brown, this study). **b**, SST difference between the North Atlantic sites with a 100-kyr moving average. Smaller differences caused by cooling indicate a reduced northward reach of the NAC. It should be noted that the moving average gradient may not capture the full variability^27^. The green line indicates the longitudinal SST difference between 3° E and 12.7° W. The yellow, black and purple lines indicate latitudinal SST differences. **c**, Relative surface salinity (orange) based on planktonic foraminifera *Globigerinoides sacculifer* δ^18^O and sand fraction accumulation rate (dark blue) with age revised^28^ at ODP site 999^34,60^. The latter indicates NADW strength. **d**, STF index indicating Agulhas Leakage, as in Fig. 2d. **e**, Global benthic foraminiferal δ^18^O stack^76^ and obliquity^77^. The pink interval and bar highlight mPWP and MIS KM5c. Darker and lighter purple highlight U^K′^_37_ and TEX_86_ divergence, MIS M2 and its deglaciation stage.

**Extended Data Fig. 1:**
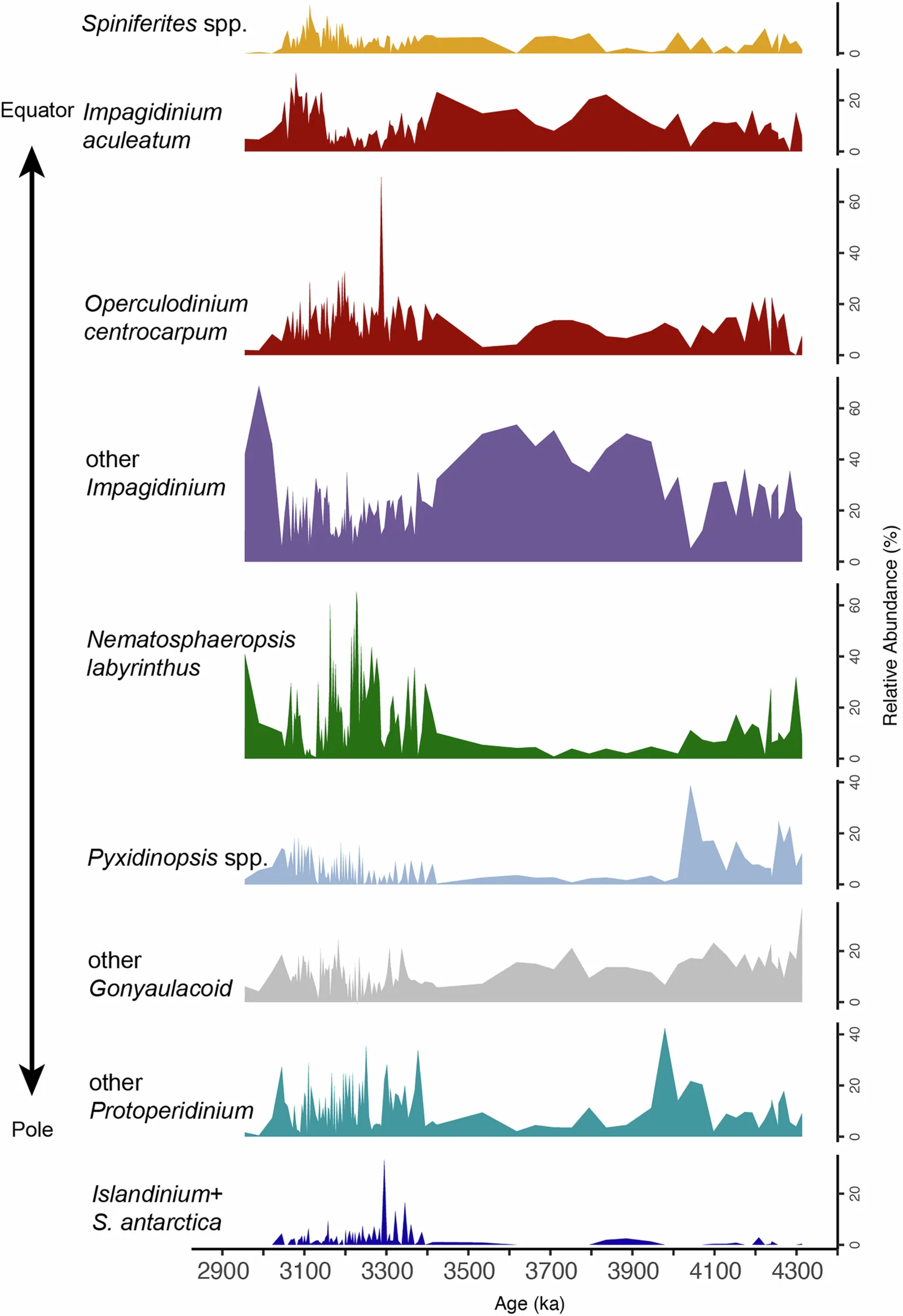
Relative abundance of dinoflagellate cyst assemblages at Site U1475 in the mid-late Pliocene. Dinocysts are grouped and ordered from equatorward (down) to poleward (up) based on their modern distribution^37^.

**Extended Data Fig. 2:**
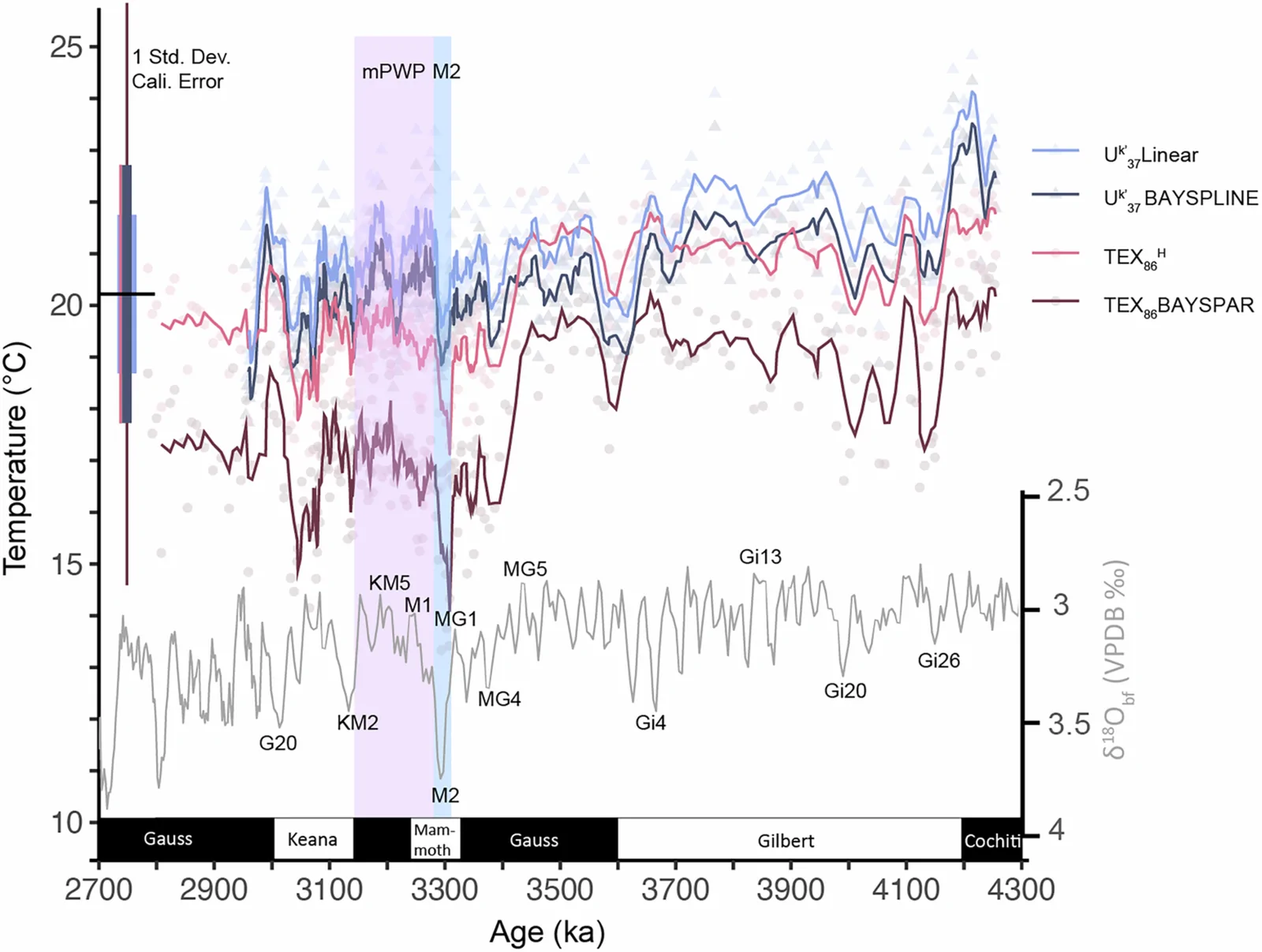
Sea surface temperature record of Site U1475 in the mid-late Pliocene. TEX_86_-based SSTs (dots) are derived from TEX_86_^H87^ and BAYSPAR^102^ calibrations respectively. U^K′^_37_-based SSTs (triangles) are derived from linear core-top^88^ and BAYSPLINE^105^ calibrations respectively. Solid lines denote 4-point moving averages. LR04 benthic δ^18^O stack^80^ is plotted on the secondary y-axis (right). Marine Isotope Stages (MIS) nomenclature follows ref. ^80^. The mid-Piacenzian Warm Period (mPWP) and Marine Isotope Stage M2 are highlighted by the pink and blue bands, respectively. Paleomagnetic reversals are based on ref. ^77^.

**Extended Data Fig. 3:**
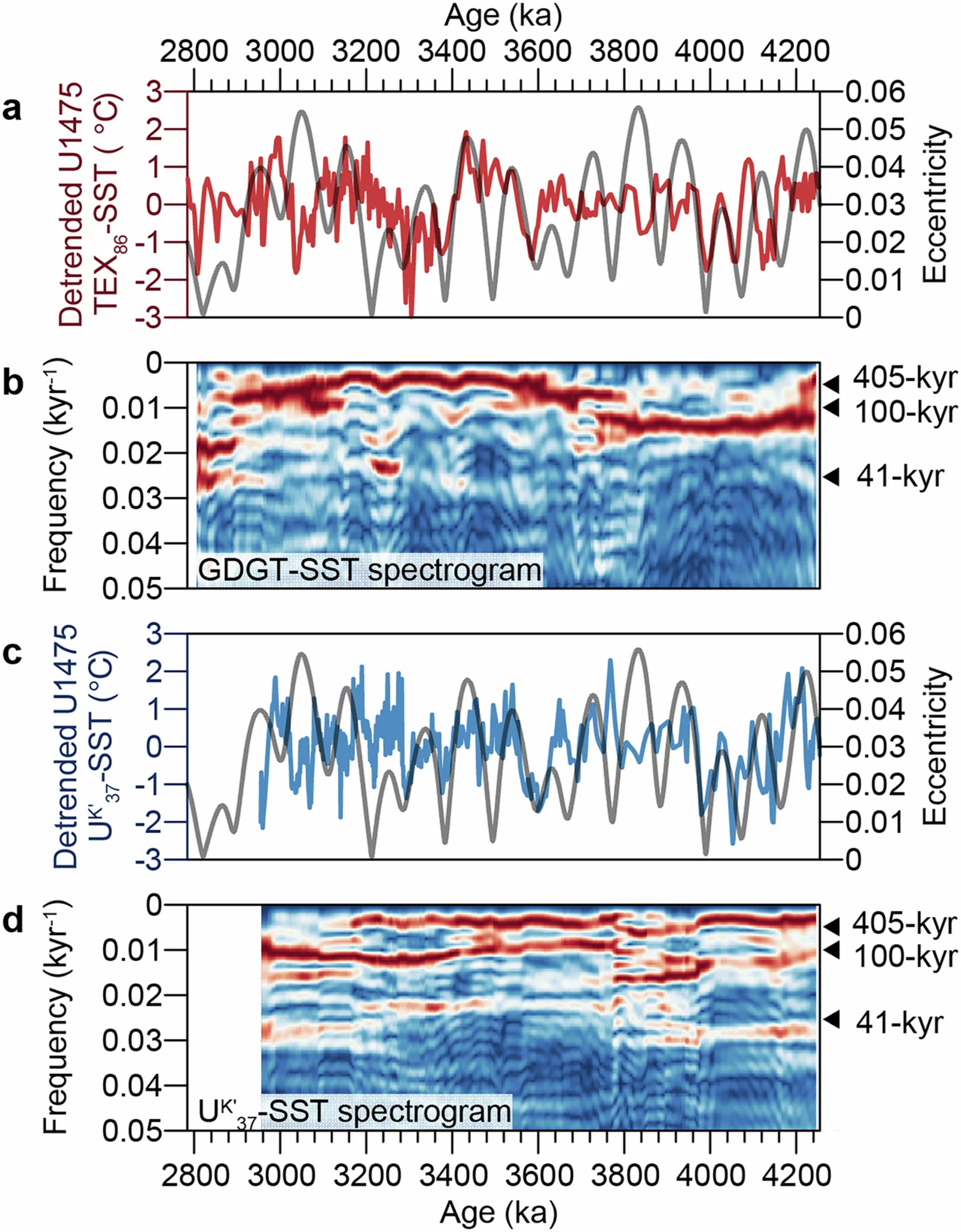
Evolutionary spectral analyses of TEX_86_ and U^K′^_37_ at Site U1475. **a**, **c**) Detrended temperature records of TEX_86_ and U^K′^_37_ respectively along with eccentricity^75^. **(b, d)** Evolutionary spectral analyses using a moving window of 405 kyr.

**Extended Data Fig. 4:**
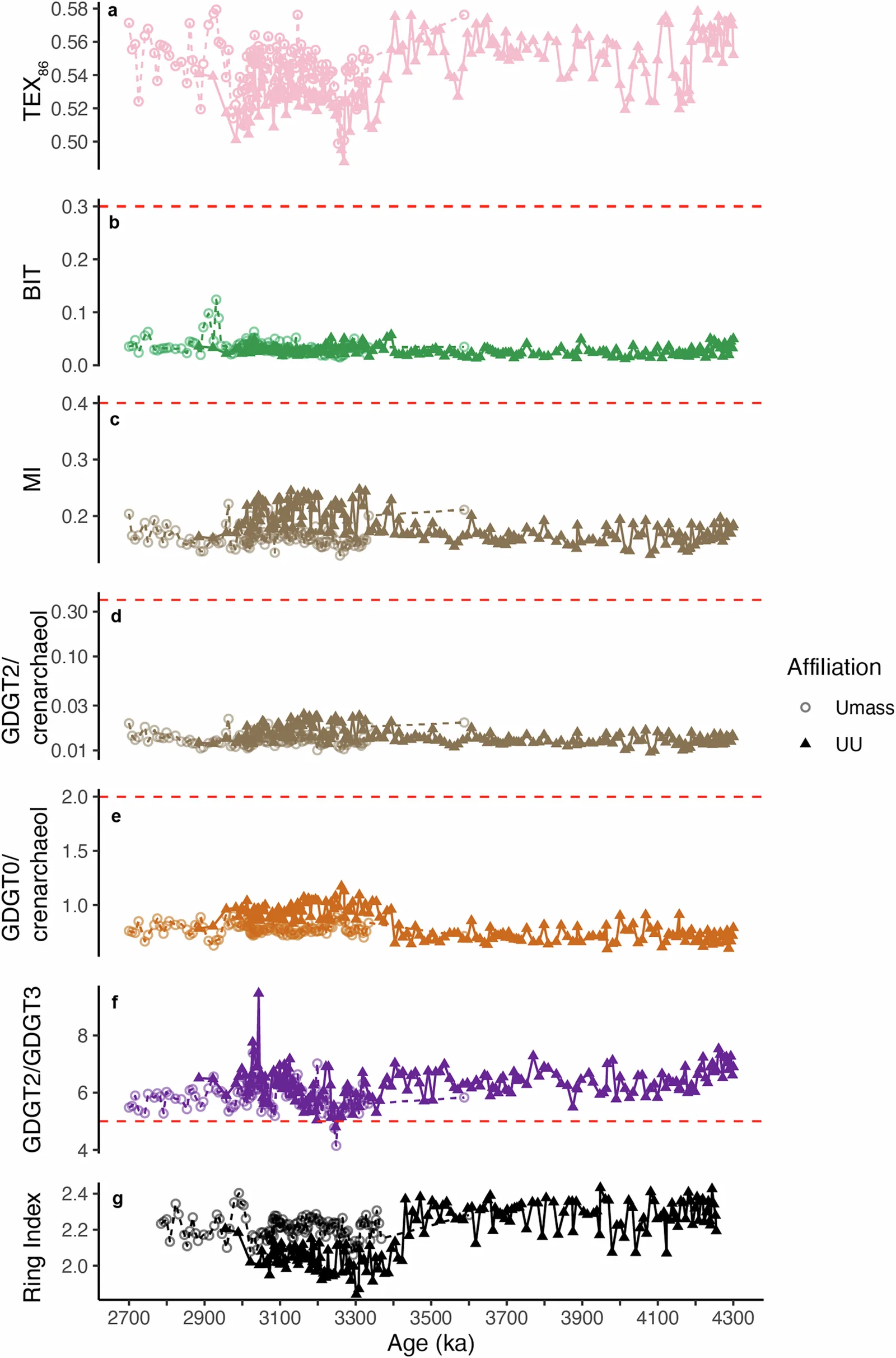
Uncorrected GDGTs indices and ratios in samples from two labs, from Site U1475. (**a**) TEX_86_^90^ (**b**) BIT, threshold=0.3^92^. (**c**) Methane Index, threshold=0.4^95^ (**d**) GDGT-2/ crenarchaeol, threshold=0.5^93^. (e) GDGT-0/crenarchaeol, threshold=2^96^. (f) GDGT-2/GDGT-3, threshold=5^50^. (g) Ring Index^98^. Red dashed lines indicate thresholds, above which a non-thermal overprint is expected on TEX_86_. Dots=UMass, triangles=UU. It should be noted that UU and UMass samples are not always from the same holes and cores from Site U1475.

Dinocyst assemblages indicate substantial and reiterative frontal migrations (Fig. 2b,d and Extended Data Fig. 1). The overall increase of *Nematosphaeropsis labyrinthus*, an indicator species for the STF^37^, suggests that the STF approached site U1475, reflected by the increase in the STF index (Fig. 2c and Methods). Between 3,400 ka and MIS M2, SSTs derived from U^K′^_37_ and TEX_86_ decline, superimposed on orbital-scale variability (Extended Data Figs. 2 and 3). Notably, TEX_86_ indicates a more pronounced cooling than U^K′^_37_, resulting in a divergence of U^K′^_37_- and TEX_86_-based temperatures from 3,400 ka onwards (Fig. 3). Given the complexity of glycerol dialkyl glycerol tetraether (GDGT) distributions and the challenges in reliably interpreting TEX_86_-based temperatures (Supplementary Discussion and Extended Data Fig. 4), our interpretation of SST evolution at this site is based on only U^K′^_37_.

The abundance of cold, present-day polar front-affiliated species (*Islandinium* spp.) (Supplementary Fig. 3) and a reduction in species found today north of STF species during MIS M2 (Fig. 2) suggest that the site was under the influence of subpolar conditions at this time. The high contributions of *Islandinium* spp. at site U1475 during MIS M2 indicate the northernmost position of the STF. In addition, the 3 °C cooling during M2, as seen in multiple records from the Agulhas region (Fig. 2a)^36,38,39^, is comparable to the degree of cooling reconstructed for Pleistocene glacial–interglacial cycles^8^, in which 3 °C cooling has been linked with ACC and STF migration by a few latitudinal degrees^40–42^ and the potential shutdown of AL^8^. The northward migration of the STF from 3,400 ka to MIS M2, inferred from dinocyst assemblages, is consistent with results from coccolithophores at the same site^43^. The subpolar conditions at U1475 were swiftly terminated during the deglaciation stage of the M2 interval (3,287 ka), as indicated by the substantial increase of the warm-temperate and relatively higher-salinity species *Operculodinium centrocarpum*^37,44,45^ (Figs. 2b and 3d).

During the mPWP, species from south and north of the STF alternate in dominance on time scales of 40 kyr (sampling resolution is ~3 kyr; Fig. 2b,c). Apart from during MIS M2, the STF reaches a northern position at ~3,240 ka and ~3,160 ka, corresponding to MIS KM6 and approximately KM2, respectively. By contrast, MIS KM5c (3,205 ka) is characterized by a more poleward-positioned STF, indicated by reduced abundance of *N. labyrinthus*, prevalence of warm-water species and increased SST (Fig. 2b).

In theory, the STF migration described above should have introduced substantial variations in AL. However, late Pliocene records from the northeastern boundary of the modern AL pathway in the Southern Benguela region (ODP site 1087) contain very few Indian Ocean planktonic foraminifera that are typically used as AL indicators^35,36^. Instead, these studies reported an extended and/or southward-shifted Benguela upwelling zone and enhanced dust input in the late Pliocene, which are interpreted as strengthened southeasterly trade winds. The discrepancy between our STF record and the lack of changes in the Benguela Upwelling region leads to two possible explanations: (1) STF migration did not lead to any AL change or (2) AL variability was not adequately captured at site 1087. The elevated Ti/Al ratios between 3,500 ka and 2,800 ka at site 1087 suggest a generally poleward-positioned Southern Hemisphere wind belt during this period^36^, whereas the relatively lower Ti/Al values during MIS M2 suggest a more northward position, in line with our dinocyst results. Although the site is in the pathway of AL today, it is difficult to exclude that the main AL pathway was shifted poleward and bypassed site 1087 in the Pliocene^3,36^.

**Extended Data Fig. 5:**
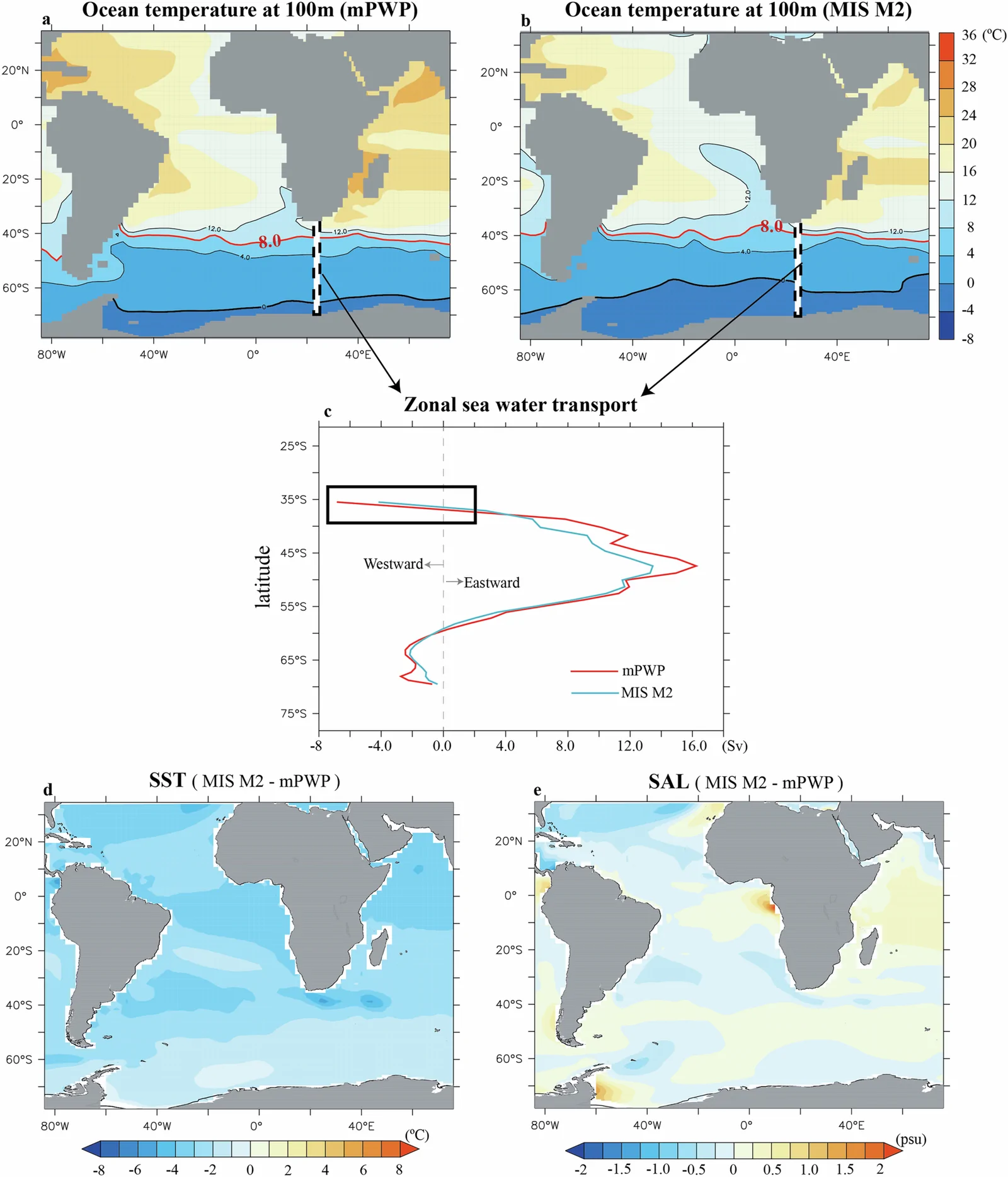
Simulated ocean surface conditions for MIS M2 and mPWP. (**a**-**b**) Subsurface ocean temperature at 100 m depth. According to the definition of the Subtropical Front (STF) by Orsi et al.^72^, the approximate location of the STF lies within a band where temperatures at 100 m depth increase from 10 °C to 12 °C. In this study, considering model biases and the likely position relative to the modern STF, we use the 8 °C isotherm as a theoretical STF position specifically for the modelling results. (**c**) Zonal ocean water mass transport through the passage between southernmost Africa and Antarctica. Positive values denote eastward transport. The rectangular area represents the region where the major Agulhas Leakage (AL) occurs. (**d**-**e**) SST and salinity differences between MIS M2 and mPWP. Figure generated with Ferret (http://ferret.pmel.noaa.gov/Ferret).

**Extended Data Fig. 6:**
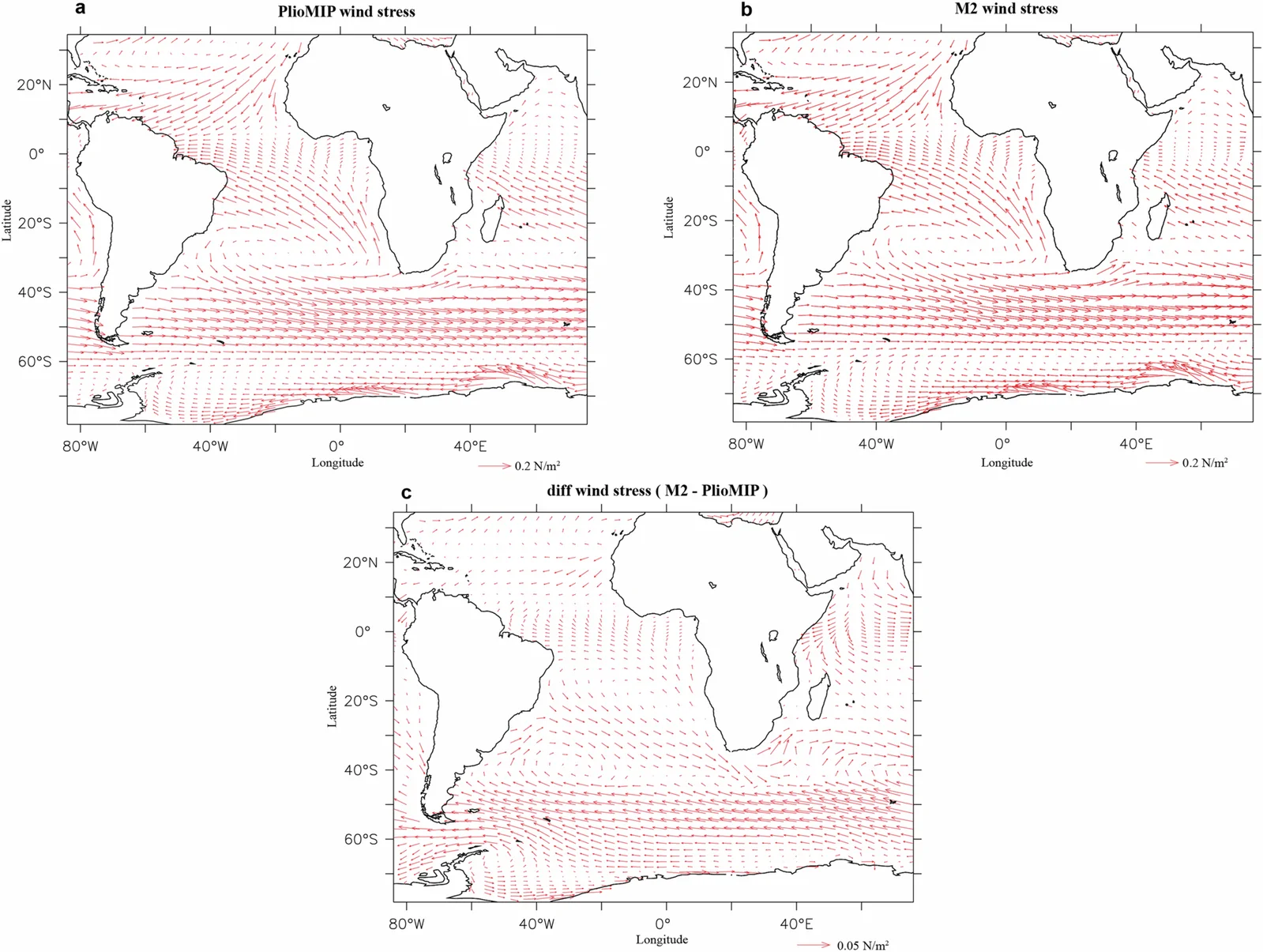
Simulated wind stress for MIS M2 and mPWP. (**a**, **b**) wind stress over ocean for mPWP and MIS M2. (**c**) differences between MIS M2 and mPWP. Figure generated with Ferret (http://ferret.pmel.noaa.gov/Ferret).

To assess from numerical model simulations whether there was a change in AL in the late Pliocene, we revisit previously published MIS M2–mPWP modelling outputs^21,46^, focusing on the Agulhas area. The decrease in SST and salinity in the Agulhas region and the weakened wind stress in the Southern Benguela region during MIS M2 than mPWP would have reflected a northward-positioned STF and a reduced AL (Extended Data Figs. 5 and 6).

## Shoaled Atlantic thermocline and strong overturning at M2

**Extended Data Fig. 7:**
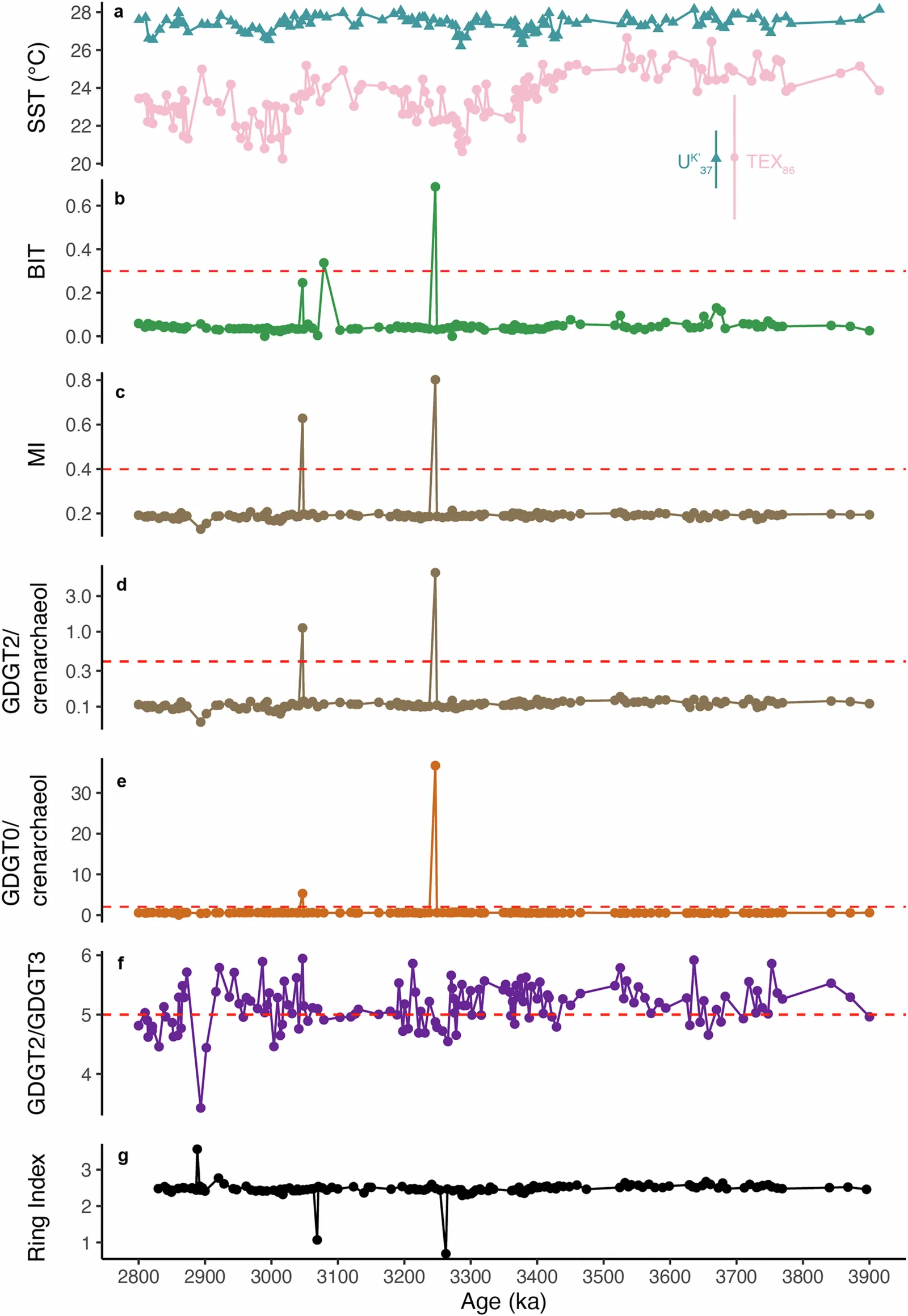
GDGTs indices and ratios of Site 625 in the late Pliocene. (**a**) TEX_86_^H^ and U^K′^_37_ based temperature with calibration error^87,88^ (**b**) BIT, threshold=0.3^92^. (**c**) Methane Index, threshold=0.4^95^. (**d**) GDGT-2/crenarchaeol, threshold=0.5^93^. (**e**) GDGT-0/crenarchaeol, threshold=2^96^. (**f**) GDGT-2/GDGT-3, threshold=5^50^. (**g**) Ring Index^98^. Red dashed lines indicate thresholds, above which a non-thermal overprint is expected on TEX_86_.

U^K′^_37_-based SST estimates in the northern GoM range from 26 °C to 28 °C (Extended Data Fig. 7). In addition, TEX_86_ shows a distinct, stepwise decline of ~3 °C centred around 3,400 ka (Fig. 3a). This pronounced cooling marks the onset of a divergence between TEX_86_- and U^K′^_37_-derived temperatures, synchronous with the divergence observed at site U1475 (Fig. 3a). Besides sites U1475 and 625, a divergence between TEX_86_ and U^K′^_37_ is also recorded at site 959 in the equatorial east Atlantic^47^ (Figs. 1 and 3a). At site 959, the thermocline shoaled at ~3,400 ka, indicated by an increase in the vertical gradient between surface- and thermocline-dwelling foraminiferal δ^18^O and Mg–Ca^47,48^. Analogously, we attribute the divergence between TEX_86_- and U^K′^_37_-derived SSTs at site 959 to increased GDGT contribution from below the shoaled thermocline (Supplementary Fig. 1)^47,49,50^. Furthermore, a reduced ring index (RI) and increased GDGT-0/crenarchaeol coincide with the thermocline shoaling at ~3,400 ka (Supplementary Discussion and Extended Data Figs. 4 and 7) and are recorded synchronously at all three sites^47^. Similar trends in the RI, together with increased TEX_86_- and U^K′^_37_-temperature difference related to thermocline dynamics, also characterize the Atlantic Ocean in the Pleistocene, although the former was not described in the original publication^51^. Consequently, we surmise that thermocline shoaling was likely pan-Atlantic. This may have been forced by changes in the water properties of the NADW, which was present at depth at all three sites^52^. As proposed by previous studies^51,53–55^, once NADW formation intensifies, the Atlantic subsurface cools owing to an enhanced supply of relatively cold water and the thinning of the upper ocean layer^56,57^, leading to thermocline shoaling. Another potential contributor to shoaling thermocline can be an intensification of intermediate water formation close to the Southern Hemisphere STF, which feeds site 959 as South Atlantic Central Water^52,58^, site 625 as Antarctic Intermediate Water and/or Subantarctic Mode Water^59^, and site U1475 locally, given the general northward migration trend of STF from 3,400 ka onwards (Fig. 2c).

**Fig. 4:**
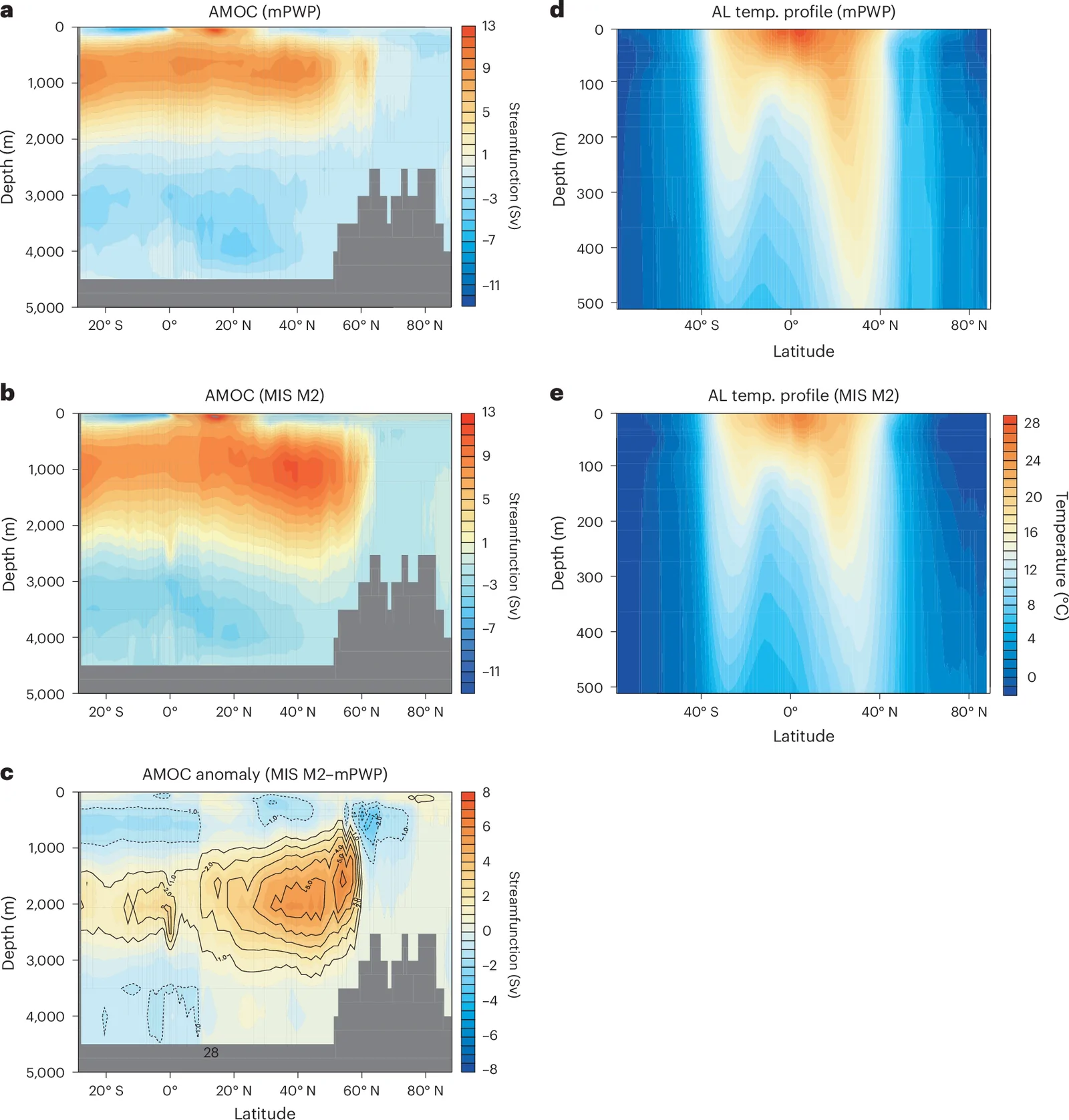
Simulated AMOC and zonal-mean upper-ocean temperature profiles of MIS M2 and mPWP along the Atlantic transect. **a**,**b**, AMOC streamfunction during mPWP (**a**) and MIS M2 (**b**). Positive values mean seawater travels clockwise along the contour. **c**, AMOC streamfunction difference between MIS M2 and mPWP. **d**, 0–500 m Atlantic Ocean temperature profile during mPWP. **e**, 0–500 m Atlantic Ocean temperature profile during MIS M2. Figure generated with Ferret (http://ferret.pmel.noaa.gov/Ferret).

**Extended Data Fig. 8:**
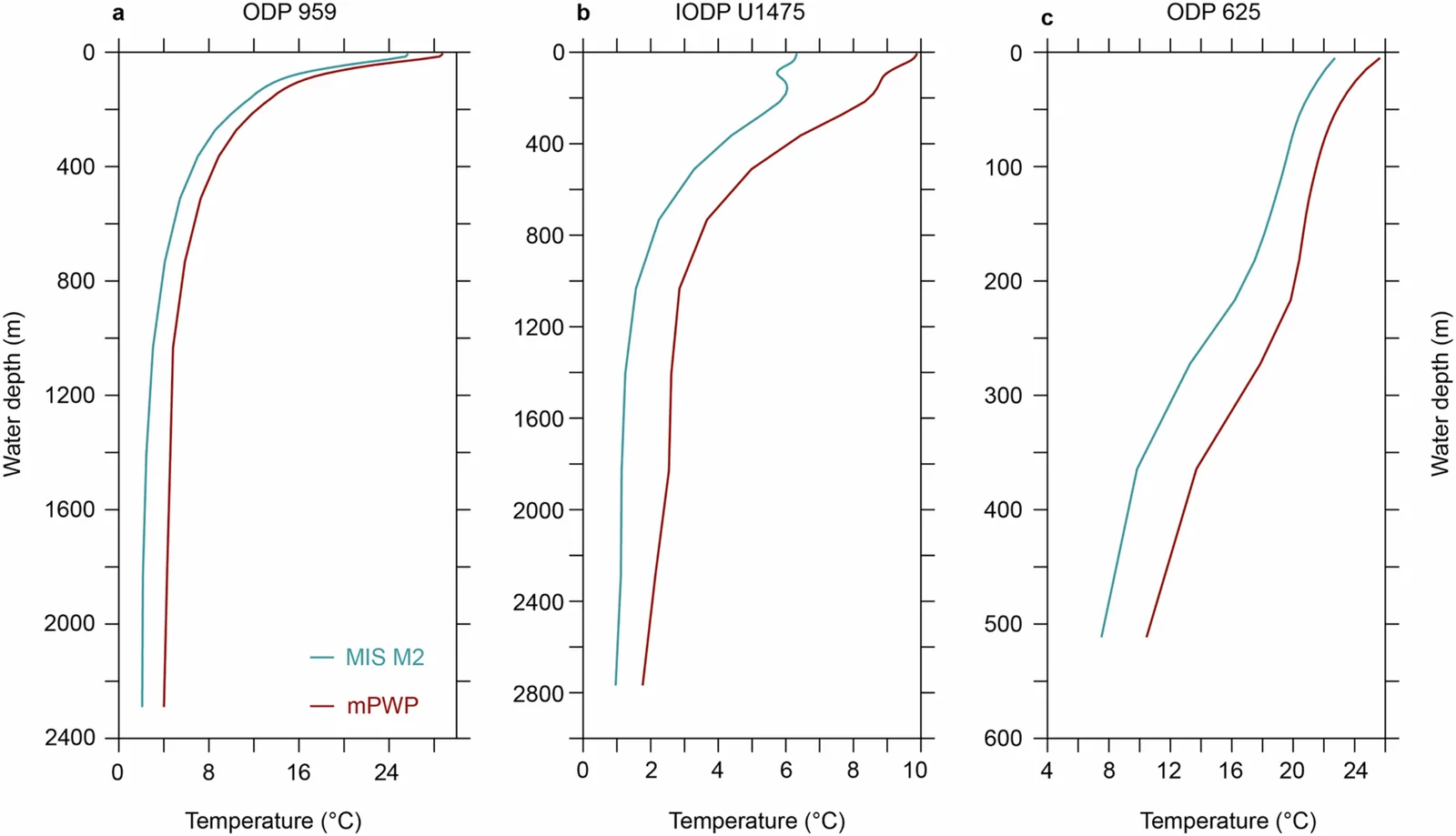
Site-specific water column temperature profile of MIS M2 and mPWP. (**a**) ODP 959, 0-2400 m water depth. (**b**) IODP U1475, 0-2800 m water depth. (**c**) ODP 625, 0-600 m water depth. Modelling outputs were processed as in Fig. 4.

Model simulation results regarding the AMOC broadly agree with the proxy-based interpretation. The simulated AMOC in MIS M2 was overall enhanced at greater depths but decreased in the surface layer and north of 58° N compared with the mPWP (Fig. 4a–c). The AMOC strength, defined as the maximum streamfunction value north of 20° N and below 500 m water depth, is about 11.78 Sv in MIS M2 and 10.94 Sv in the mPWP. Furthermore, simulations indicate a noticeably shoaled warm-water layer in the upper 500 m during MIS M2, compared with the mPWP (Fig. 4d,e), which is also seen in site-specific water-column temperature profiles (Extended Data Fig. 8).

## Disconnected AL and AMOC dynamics

**Fig. 5:**
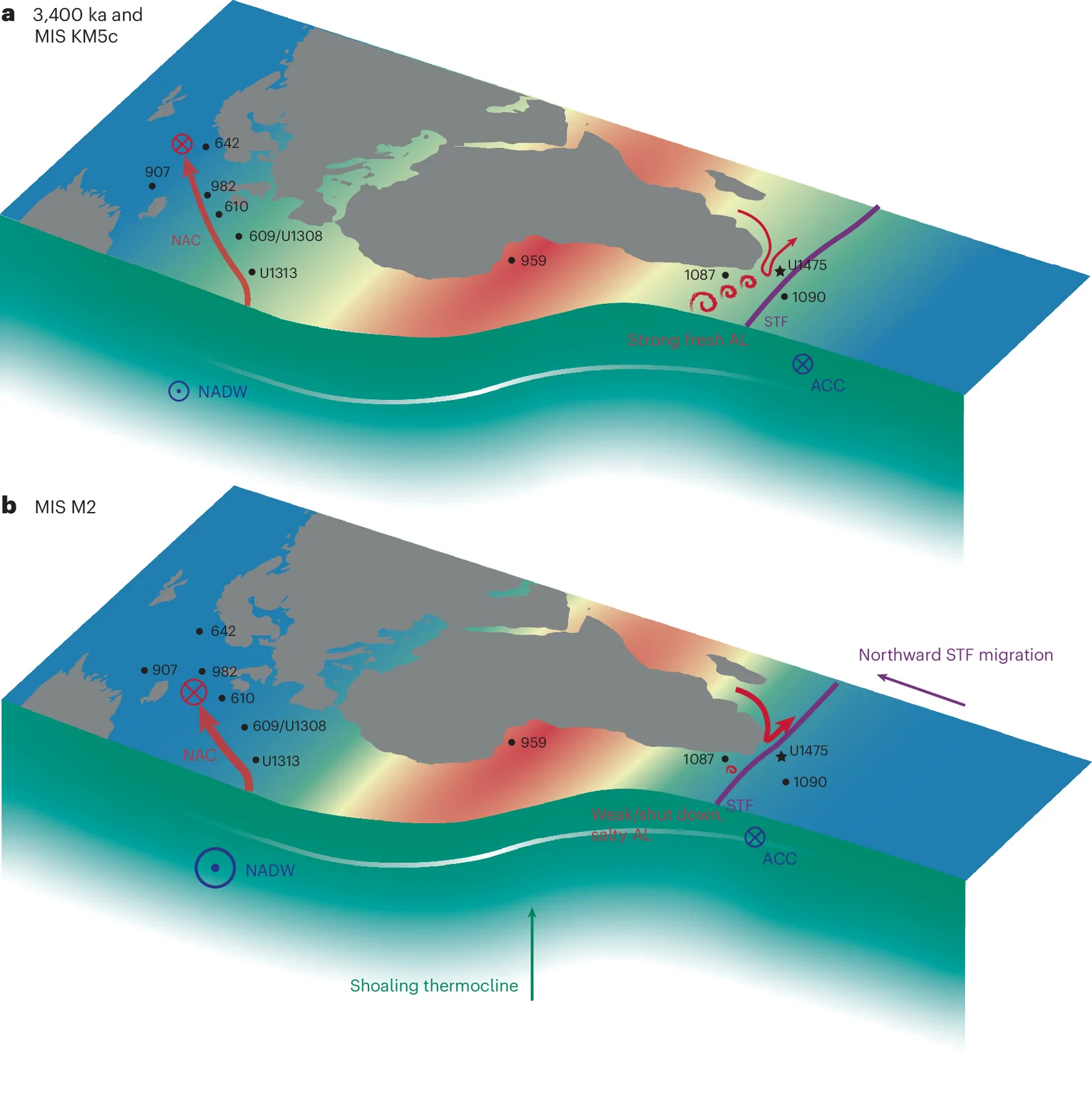
Conceptual oceanographic changes of the AL, NADW and NAC across MIS M2. **a**, At 3,400 ka and during MIS KM5c, the STF was southerly positioned, allowing a strong, low-salinity AL into the Atlantic. NADW formation happened far north, and a large latitudinal SST gradient persisted in the North Atlantic. **b**, During the MIS M2 peak glaciation, the STF moved equatorward and possibly shut down the AL. A weaker but slightly saltier AL entered the Atlantic. The NAC did not reach far north and sank at lower latitudes; subsequently, North Atlantic SSTs dropped, and its latitudinal gradient collapsed. Intensified NADW formation thus happened at lower latitudes and shoaled the Atlantic thermocline. The reach of the NAC and location of NADW formation are based on Fig. 4 and ref. ^28^. Colours of the sea surface indicate SST. The vertical profile, represented by the teal-white shading, indicates the water column, and the white shading line indicates the thermocline. The thickness of the arrows indicating currents reflects salinity. It should be noted that all colours and patterns are for descriptive purposes only; thus, there is no quantitative scale. Visualization is based on this study and refs. ^27,61^. Basemap in **a** created with tmap^74^.

In theory, the diminishing AL from 3,400 ka to MIS M2 should lead to a lower salinity in the AMOC loop, thereby weakening AMOC. Several sites (IODP sites 982, U1313, 642 and 610) along the NAC pathway show a decrease in SST and a simultaneous increase in the SST gradient between sites U1313 (40° N) and 610 (53° N)^27,29–31^ (Figs. 3b and 5) from 3,400 ka to MIS M2. This trend was interpreted as reflecting reduced NAC influence on these high-latitude sites (Figs. 3b and 4)^27,29,31–33^ and an Arctic front position south of these sites^29,33^. Simultaneously, the salinity in the Caribbean Sea increased, indicated by planktonic foraminifer δ^18^O^60^ and dinocyst assemblages^28^. Even if AL ultimately contributed to Caribbean Sea salinity, we consider it unlikely that the changes in AL salinity compensated for the reduction of AL during phases of northward STF migrations. Taking the Pleistocene as a reference ^61^, the salinity of AL waters is set by Indian Ocean salinity changes, which in turn relates to changes in the Indonesian Throughflow (ITF). However, it is unlikely that this had a role at the higher sea level of the Pliocene. An existing ITF record also indicates a relatively fresher ITF between 3,500 ka and the onset of MIS M2 than before or after^62^, implying that Indian Ocean salinity variability was not comparable to its Pleistocene amplitude and unlikely relevant to the salinity of the Caribbean Sea in the late Pliocene through AL.

**Extended Data Fig. 9:**
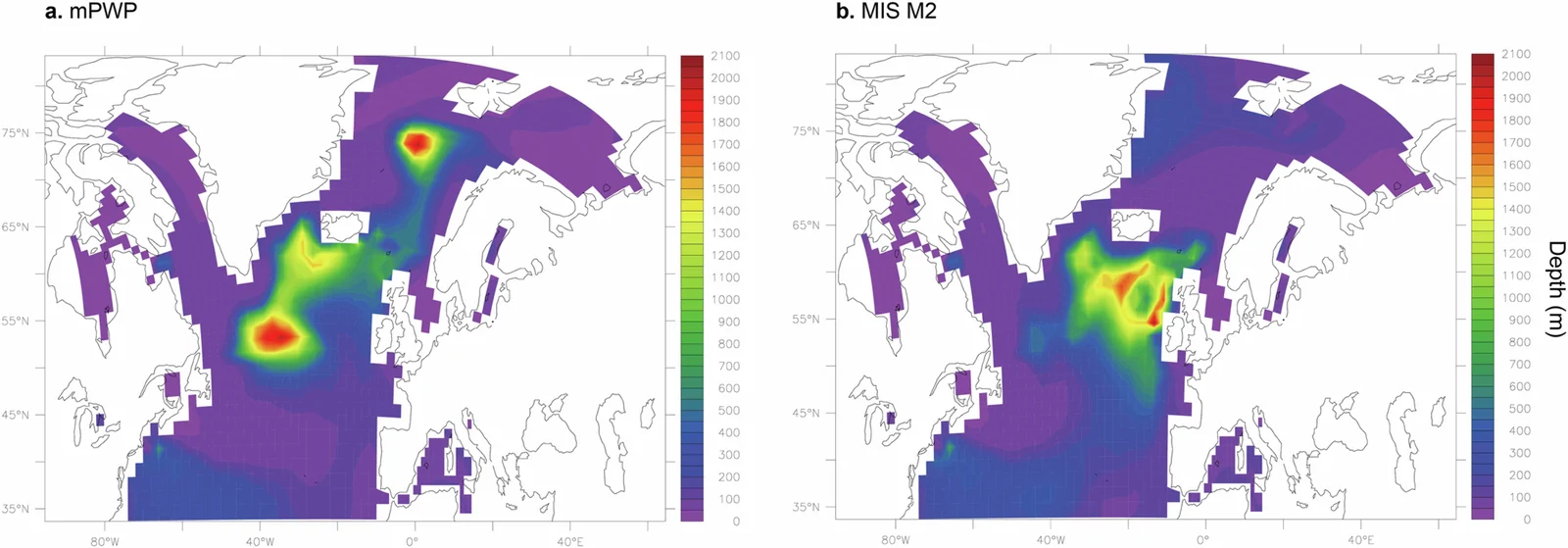
Maximum winter mixed layer depth. (**a**). mPWP **(b)**. Figure generated with Ferret (http://ferret.pmel.noaa.gov/Ferret).

Counterintuitive with respect to diminished NAC influence in the Norwegian Sea are signs of an intensification of NADW formation around 3400 ka, inferred from increasing carbonate-sand fraction in Caribbean Sea sediments (Fig. 3c)^34,60^ and a diminished benthic δ^13^C gradient along the Atlantic transect^63^. Conspicuously, this strengthening of NADW formation is concomitant with the thermocline shoaling recorded at sites U1475, 959 and 625 (Figs. 3 and 5b). Thus, the outstanding question is: how does a decrease in the northward reach of the NAC relate to an increase in the strength of the lower limb of the AMOC and thermocline shoaling at several Atlantic locations? Taking modelling outputs and proxy-based reconstructions together, modelling outputs suggest a weakened NAC influence in the high-latitude Atlantic alongside an intensified NADW formation during MIS M2 compared with warm climate conditions (Fig. 4a,b), consistent with palaeoceanographic evidence presented in previous studies mentioned above^28,29,34^. There is also a substantial streamfunction reduction at 60°–70° N in the upper 2,000 m of the water column, pointing to a basin-average southward shift of NADW formation, at least from the Nordic Sea, indicated by the location of maximum winter mixed-layer depth (Extended Data Fig. 9). The Atlantic overturning at lower latitudes becomes even stronger during MIS M2 than in the surrounding warm periods (Figs. 4b and 5)^20^. Modelling outputs also reproduce pan-Atlantic thermocline shoaling (Fig. 4d and Extended Data Fig. 7), consistent with the thermocline shoaling when NADW formation intensifies^56,57^.

In the deglaciation phase of MIS M2, dinocyst assemblages at site U1475 record an abrupt southward STF migration, implying a revival of AL (Fig. 2). Besides the prevailing *Operculodinium*, the high-salinity species *Tuberculodinium vancampoae* is sporadically present in the same sample^45^. The presence of this warm, lagoonal species could suggest episodic release of relatively saline Indian Ocean water into the Southern Atlantic through AL during M2 deglaciation. A similar scenario has been proposed for the Pleistocene, in which salinity built up in the Indian Ocean during glacial phases is released into the Atlantic during deglaciations^61^. This scenario could also be true for MIS M2, although the overall high Pliocene sea levels^64^ may have complicated this. However, our record does not provide evidence for the proposed link between AMOC and the release of saline Indian Ocean water during the deglaciation phase of MIS M2^61^. Notably, the inferred increased salinity of AL during the MIS M2 deglaciation does not seem to be conveyed to the Caribbean Sea, where decreased planktonic foraminifer δ^18^O instead indicates freshening^60^ (Fig. 3c). Furthermore, no abrupt change in NADW strength can be observed in the Caribbean Sea grain-size record (Fig. 3c). Therefore, STF migration and subsequent variations in AL strength appear disconnected from the AMOC strength during the deglaciation of MIS M2 as well.

During the mPWP, including 3,205 ka (MIS KM5c), dinocyst species typically found north of the STF are abundant in our records, indicating that the STF reached its southernmost location, implying a potential maximum in AL. Our reconstruction is consistent with the findings from the modelling simulations, which suggest that a poleward shift of the STF and Westerlies during the mPWP is plausible (Extended Data Fig. 6)^65^. Regarding the AMOC, palaeoceanographic records and available modelling results^20,21^ suggest a further northern reach of the NAC^27,28,32^, but a fresher Caribbean Sea^60^ along with weaker NADW formation^34^ during the mPWP relative to MIS M2 (Figs. 3c, 4b and 5c).

There is now an increasing body of work suggesting that, rather than AL, local North Atlantic buoyancy forcing exerts a direct major influence on AMOC strength in the Quaternary^66,67^. The exact architecture of Arctic gateways governs low-salinity water entering the North Atlantic and therefore has an important role in AMOC, especially in the mPWP simulation ensemble^23,24^. However, the available simulation ensemble does not enable a comparison of AMOC with MIS M2. Given the uncertainties in these geological settings^25,26,68^, we can hardly support an implication that conditions of Arctic gateways changed concurrently during the late Pliocene. Available ice-rafted debris records^69,70^ suggest that meltwater was only possible to be prevalent during MIS M2, which should, in theory, reduce the AMOC strength together with diminished AL. With the Greenland ice sheet being smaller in the late Pliocene^71^ than today, the buoyancy forcing likely came from other sources: high-latitude precipitation and sea ice boundary, for instance.

In summary, we find no apparent relationship between AL and AMOC during the late Pliocene. In addition, the divergence in TEX_86_- and U^K′^_37_-based SST records around 3,400 ka, recorded at multiple sites along the AMOC pathway, reveals a pan-Atlantic thermocline shoaling. We therefore propose that a southward shift in the NADW formation locus is concomitant with AMOC intensification and the pan-Atlantic thermocline shoaling. However, these AMOC dynamics are independent of AL but more likely dependent of local factors in the North Atlantic^24^. Our data imply that the majority of AL either did not reach the Caribbean Sea and North Atlantic^15^ or did not cause an essential salinity change to regulate AMOC, and thus challenge the long-held hypothesis^1^ that AMOC dynamics are (partly) forced by AL^7,8,11^, at least in the late Pliocene.

## Methods

### Site description

IODP site U1475 (41° 25.61′ S, 25° 15.64′ E; 2,669 m water depth) was drilled on the southwestern flank of the Agulhas Plateau, at the intersect of the Southern, Indian and Atlantic Oceans (Fig. 1)^78^. Today, site U1475 is located between the ARC and the STF, with seasonal SSTs between 11 °C and 17 °C (ref. ^79^). The site is currently bathed by the NADW^78^. Thus, it records the history of not only the latitudinal migration of the STF but also consequent SST changes in the geological past^78^. We use sediments retrieved from site U1475 holes B, C and F as a continuous splice of composite depth (CCSF) between 70 m and 140 m CCSF.

ODP site 625 (28° 49.9′ N, 87° 09.6′ W; 889 m water depth) was drilled in GoM, which is under the influence of NADW^59^, during ODP leg 100. Site 625 is on the path of warm Caribbean waters, which subsequently feed the Gulf Stream and the NAC (Fig. 1). Annual SST at site 625 ranges from 21 °C to 29 °C^79^. We use the sediments retrieved from site 625 hole B between 129 m and 165 m below the sea-floor.

### Age models

The age model of site U1475 used in this study follows ref. ^43^. The shipboard age model^78^ was refined by aligning the shipboard physical properties records^80^ with the LR04 global benthic foraminiferal^81^ and probabilistic^82^ δ^18^O stacks (Supplementary Fig. 2 and Table 1). We linearly interpolate sample ages using LOESS smoothing. The late Pliocene interval of site 625 was dated using benthic foraminiferal δ^18^O stratigraphy by calibrating to the LR04 global benthic foraminiferal stack^81^ by ref. ^83^ (Supplementary Fig. 2).

### Palynology and a dinoflagellate cyst-based STF migration reconstruction

A total of 112 sediment samples from site U1475 were processed for palynology, focusing on dinoflagellate cyst assemblages. The mPWP interval has a higher sampling resolution (average 4 kyr per sample) than the rest of the record (average 30 kyr per sample). Approximately 9 g of sediment was freeze-dried and subsequently crushed. A known number (*n* = 9,666 or 19,855) of *Lycopodium clavatum* spores was added to each sample for quantification. Hydrochloric acid (30%) and cold hydrofluoric acid (38%) were added to remove carbonates and silicates, respectively. The remaining residues were then sieved through a 10-μm nylon mesh, with an ultrasonic bath to disaggregate agglutinated organic particles. Subsequently, the residues were mounted on glass slides using glycerine jelly, sealed with varnish and counted using an Olympus CX41 optical microscope (under ×200 and ×400 magnification). At least 200 dinocyst specimens were counted per sample, if possible. No samples yielded fewer than 50 dinocyst specimens. Terrestrial and other aquatic palynomorphs were also counted but not determined to species level^45^.

We apply a recent overview of modern Southern Ocean dinocyst distributions^37^ to reconstruct mid-Pliocene surface ocean conditions at site U1475. K-means cluster analysis grouped Southern Ocean surface sediment samples into nine clusters, each with one dominant dinocyst species. We use the observations in ref. ^37^ that samples in these clusters are strongly geographically separated latitudinally, tied to specific ocean zones in the Southern Ocean. Specifically, these clusters reflect the strong latitudinal gradients in SST, sea ice existence, salinity and nutrient conditions. In particular, the cluster with dominant *N. labyrinthus* is characteristic of the Subantarctic Zone, south of the STF; those with dominant *Impagidinium aculeatum* (Iacu-cluster), *O. centrocarpum* (high-ocean cluster) and *Spiniferites* spp. (spin cluster) thrive north of the STF; *Protoperdinium* cysts are considered as a high-productivity indicator and dominate in samples from around the polar front (high-brig cluster); *Islandinium* spp. (Imin cluster) indicate the presence of (summer) sea ice and occur particularly around the Antarctic Peninsula today. Supplementary Fig. 3 provides an overview of the latitudinal distribution of dinocyst assemblage clusters in the modern ocean. The latitudinal position of the STF is then inferred from an STF index, based on the relative abundances of dinocyst taxa south of the STF (south of STF/(south + north of STF)) to show the migration of the STF. Although it does not directly indicate the exact latitude of the STF, a higher value indicates that the STF is positioned relatively further north and therefore, in theory, has less AL, and vice versa.

### Biomarker analyses

A total of 183 sediment samples from site U1475, collected roughly every 15 cm from the splice, were processed for lipid analysis in the GeoLab at Utrecht University (UU). About 6 g of sediment was freeze-dried and homogenized before extraction with 25 ml of 9:1 (*v*/*v*) dichloromethane (DCM)–methanol (MeOH) using a Milestone Ethos X microwave system at 70 °C for 30 min. Sediments from ODP site 625 (*n* = 137) were extracted with DCM–MeOH (9:1, *v*/*v*) using accelerated solvent extraction (ASE 350, Dionex) in the GeoLab at UU. The ASE extraction method consisted of three 5-min extraction cycles at 100 °C and 7.6 × 10^6^ Pa.

C_46_ standard (99 ng) was added to the total lipid extracts to quantify the absolute concentration of GDGTs. Activated Al_2_O_3_ columns were used to separate the total lipid extract into three fractions, using hexane–DCM (9:1, *v*/*v*), hexane–DCM (1:1, *v*/*v*) and DCM–MeOH (1:1, *v*/*v*) for apolar, ketone and polar fractions, respectively. Polar fractions were dried under a gentle N_2_ stream, then redissolved in hexane–isopropanol (99:1, *v*/*v*) and filtered using a 0.45-μm polytetrafluoroethylene filter before analysis on an Agilent 1260 Infinity series HPLC system coupled to an Agilent 6130 single-quadrupole mass spectrometer, following instrumental and analytical set-up as described in ref. ^84^.

At UU, the ketone fractions were dissolved in ethyl acetate and analysed on a gas chromatograph (GC) coupled to a flame ionization detector (GC-FID; Hewlett Packard 6890 series) equipped with a CP-Sil 5 fused-silica capillary column (25 m × 0.32 mm; film thickness 0.12 µm) and a 0.53-mm precolumn. The fractions were injected on-column at 70 °C with helium as a carrier gas at a constant pressure of 100 kPa. The oven programme was as follows: 70 °C for 1 min, then ramped to 130 °C at 20 °C min^−1^, then to 320 °C at 4 °C min^−1^, and finally held isothermally for 10 min. Di- and tri-unsaturated C_37_ alkenones were identified based on retention time, and a known amount of squalane was used as an internal standard for quantification.

A second set of samples from site U1475 was analysed for GDGTs (*n* = 128) and alkenones (*n* = 302) at the University of Massachusetts Amherst (UMass) and the University of Notre Dame (ND), respectively. At UMass, GDGTs were analysed using an Agilent 1260 series HPLC coupled to an Agilent 6120 single-quadrupole mass detector. The instrument was set up according to ref. ^84^. At ND, alkenones in the ketone fractions were identified and quantified using a Thermo Fisher Scientific Trace 1310 GC-FID coupled to an ISQ MS system, using 5α-androstane as an internal standard. The Trace 1310 GC was equipped with an Rtx-5 column (60 m × 0.25 mm × 0.25 μm) with a temperature programme starting at 70 °C, then increasing by 20 °C min^−1^ to 130 °C, and finally, increasing by 4 °C min^−1^ up to 320 °C, where it was held for 20 min^85^.

### Interlaboratory comparison to integrate SST records for site U1475

As mentioned above, biomarker data were generated in different laboratories, including UU, UMass and ND, which warrants an assessment of the interlaboratory consistency and the fidelity of merging the results from different instruments.

A first compilation of the biomarker data from IODP site U1475 indicated that the records from different laboratories are consistent in timing and direction of trends. However, the proxy values from UMass and ND are approximately 0.03 (TEX_86_) and 0.05 (U^K′^_37_) higher than those from UU (Supplementary Fig. 4). Such offsets are well within the ranges reported in global interlaboratory comparisons for TEX_86_ (0.023–0.053, translating into 1–3 °C)^86^, or the U^K′^_37_ (2.1 °C)^87^, and can be attributed to differences in instrument settings and maintenance. Furthermore, when converting TEX_86_^88^ and U^K′^_37_^89^ into SSTs, we note that, irrespective of the laboratory, U^K′^_37_-based SSTs^89^ are consistently 2–3 °C higher than TEX_86_-based SSTs (Supplementary Fig. 5)^88^.

However, to merge the data from different laboratories into one temperature record for each biomarker proxy, a subset of 15 sediment samples (randomly selected from the samples measured at UU) was also measured in the two other laboratories. This exercise showed that TEX_86_ values from UMass are consistently 0.01–0.04 (median = 0.024, average = 0.023, s.d. = 0.0085) higher than those from UU (Supplementary Table 2), comparable to the offset between the two TEX_86_ records generated at these laboratories (Supplementary Fig. 5). Hence, the TEX_86_ data generated at UMass were adjusted to the UU-TEX_86_ record by subtracting 0.024 TEX_86_ units. Unlike in the separate U^K′^_37_ records (Supplementary Fig. 4), U^K′^_37_ values show negligible difference (median = −0.0004, average = 0.0024, s.d. = 0.0068) between ND and UU in the comparison (Supplementary Table 3). Given that the U^K′^_37_-based SSTs are consistently higher than TEX_86_-based SSTs in the composite records (Supplementary Fig. 5), the U^K′^_37_ data generated at ND were adjusted to the UU-U^K′^_37_ record by subtracting 0.05 U^K′^_37_ units to resolve the temperature difference and generate a composite U^K′^_37_-SST record as shown in Fig. 2 and Extended Data Fig. 1. Thus, even though the data were generated from sediment cores retrieved from different holes of site U1475 and by different people in different laboratories, we were able to generate ‘one’ record for this site. Collectively, it should be noted that such interlaboratory offsets are unfortunately quite common^86,90^. Therefore, comparisons between records generated by different laboratories should be interpreted with caution, as should model-palaeo data comparisons, especially when the difference is subtle.

### SST reconstructions

TEX_86_ values are derived from the relative amount of cyclopentyl moieties in GDGTs^91^, which are produced by Nitrososphaerota (previously Thaumarchaeota)^92^. Several indices and ratios have been developed to identify potential contributions to the sedimentary GDGT pool from other types of archaea and are examined here (Extended Data Figs. 3 and 5). In short, the branched and isoprenoid tetraether (BIT) index is proposed to indicate a contribution of terrestrial organic matter input^93^, in which the terrestrially produced isoGDGTs^94^ can alter the TEX_86_ in marine sediments. However, BIT index values should be interpreted cautiously because in situ production of branched GDGTs in coastal marine settings can alter the BIT index itself^95^. The methane index (MI)^96^ and the ratio of GDGT-0/crenarchaeol^97^ might indicate inputs by anaerobic methane-metabolizing archaea or methanogenic archaea, respectively, although not in every setting^98^. The RI is a more general indicator of GDGTs from non-Nitrososphaerotal sources or the impact of factors other than temperature^99^. Thus, downcore changes in the RI likely indicate shifts in the archaeal community in the water column. The provenance depth in the water column of GDGTs in marine sediments is a topic of attention in recent marine isoGDGT calibration studies^47^. It is clear that Nitrososphaerotal GDGTs are predominantly produced in the water-column subsurface, at depths with maximum ammonia production below the mixed layer and near the thermocline^49,100^. However, deeper-dwelling archaea can also produce GDGTs that do not relate to surface temperature in the same way. Their contribution can be qualitatively traced by the ratio between GDGT-2 and GDGT-3^49,50,101^. Available core-top TEX_86_-temperature calibrations calibrate TEX_86_ either to the modern SST, given the strong correlation that usually exists between SSTs and thermocline temperatures, or to an assumed export depth, thus providing a subsurface calibration^102–104^. TEX_86_ values are converted to SSTs using an exponential regression^88^. A comparison of the results using this calibration with those of ref. ^103^ can be found in Extended Data Fig. 1. As TEX_86_ does not deliver a reliable SST signal at both sites (Supplementary Discussion), we do not favour either calibration.

U^K′^_37_ is based on the ratio between the di- and tri-unsaturated alkenones^105^. U^K′^_37_ is subsequently converted into SST using a core-top linear calibration^89^, which is consistent with most published data, thus enabling inter-site comparison. A comparison with another calibration^106^ can be found in Extended Data Fig. 1. The difference in reconstructed SST between calibrations is very small.

### Evolutionary spectral analyses

For spectral analysis, the merged U^K′^_37_- and TEX_86_-based SST records were smoothed with a 4-point moving average and interpolated to uniform spacing of 1.5 kyr and 3.3 kyr, respectively. The evolutionary spectral analyses were computed with a 405-kyr sliding window on the interpolated and then detrended records of U^K′^_37_- and TEX_86_-based SST. All the data preparation and analyses were conducted with Acycle^107^.

### Climate modelling

Climate simulations used in this work are derived from ref. ^21^ and were run with the Institut Pierre–Simon Laplace atmosphere–ocean coupled general circulation (IPSL-CM5A) model. More details about the IPSL-CM5A model can be found in ref. ^108^. In their work, the simulation ‘mPWP’ was performed based on the PRSIM3D boundary conditions^109–111^ and was the core simulation for the PlioMIP phase 1. ‘MIS M2’ was performed based on the equilibrated state of ‘mPWP’, by considering different forcing representing the M2 glacial, typically with a lowering of pCO_2_ (from 400 ppm to 220 ppm), the minimum summer insolation (around 3,313 ka) and the expansion of land ice sheet. More details of the experimental set-up can be found in ref. ^21^.

## Online content

Any methods, additional references, Nature Portfolio reporting summaries, source data, extended data, supplementary information, acknowledgements, peer review information; details of author contributions and competing interests; and statements of data and code availability are available at https://doi.org/10.1038/s41561-026-02055-5.

## Supplementary information

Supplementary Information Supplementary Discussion, Figs. 1–5 and Tables 1– 3.

Peer Review File

## Data Availability

All new data are available via Zenodo at https://doi.org/10.5281/zenodo.15906076 (ref. ^45^). Modelling outputs used in this study^21^ are available via Zenodo at https://doi.org/10.5281/zenodo.20379908 (ref. ^46^).

## Code availability

Due to the update of the model version, the code of IPSL-CM5A is not publicly available now and might be available on request from the IPSL group. The general information for this model can be found in the IPSL Climate Modelling Centre Wiki page (https://cmc.ipsl.fr/ipsl-climate-models/ipsl-cm5/; last accessed 26 May 2026) and in ref. ^104^.
