# Supplementary material for: Disconnection of the late Pliocene Agulhas Leakage from Atlantic Meridional Overturning Circulation
Source: Nat Geosci. 2026 Aug 3;19(10):1158–66. doi: 10.1038/s41561-026-02055-5 (PMC13645622; doi:10.1038/s41561-026-02055-5)
Supplement: Supplementary file 1 [file 41561_2026_2055_MOESM1_ESM.pdf]

nature geoscience

Supplementary information

https://doi.org/10.1038/s41561-026-02055-5

Disconnection of the late Pliocene Agulhas Leakage from Atlantic Meridional Overturning Circulation

In the format provided by the authors and unedited

This PDF file includes:

Supplementary Discussion
Supplementary Figure. S1 to S5
Supplementary Tables S1 to S3

Supplementary Discussion

Quality assurance of GDGTs for SST reconstruction and implications

Site U1475

Isoprenoid Glycerol Dialkyl Glycerol Tetraethers (isoGDGTs) dominate over branched GDGTs, indicating dominant marine sourcing (Extended Data Fig. 3b). For isoGDGTs, all confounding factor values are well below their cut-off thresholds, except GDGT-2/GDGT-3 (Extended Data Fig. 3), which is around 7, indicating some GDGTs contribution from the subsurface [1–3]. TEX_{86} values range between 0.5 and 0.6, corresponding to a temperature range of 17–23°C using the TEX_{86}^{H} calibration [4]. SSTs derived from the BAYSPAR calibration are consistently 3–4°C lower than those from TEX_{86}^{H} (Extended Data Fig. 1). TEX_{86}^{H}-based SST shows a substantial drop of 3 °C at ~3400 ka (Extended Data Fig. 1). The Ring Index and GDGT-0/crenarchaeol display a concurrently stepwise change (Extended Data Fig. 3), although they remain below reported cut-off values that would signal overprints. The change is mostly due to a stepwise increase of GDGTs with less cyclopentane moieties, especially GDGT-0. The relative increase of GDGT-0 could indicate a shift in the archaeal community and/or the export depth of GDGTs in the water column, resulting from a reorganization of the water column structure [3,5]. TEX_{86} reflects a deeper water mass temperature after 3400 ka associated with thermocline shoaling, compared to before based on the simultaneous stepwise change in Ring Index and GDGT-0/crenarchaeol. This implies that TEX_{86} should be interpreted with caution in terms of absolute SST. Our interpretation of SSTs in the discussion is, therefore, based on $U^{K'}_{37}$-derived SSTs only.

Orbital cyclicity near the eccentricity bandwidth dominates the TEX_{86} record, wherein the main cyclicity shifted from the short eccentricity ~100 kyr to the long eccentricity ~405 kyr cycle around 3700 ka. Obliquity-pacing is only detected intermittently around 3300–3200 ka and 2900–2800 ka (Extended Data Fig. 2). In the $U^{K'}_{37}$ record, significant frequencies correspond to both eccentricity and obliquity throughout the record (Extended Data Fig. 2), which resembles the pattens of local annual insolation. The difference in orbital pacing between TEX_{86} and $U^{K'}_{37}$ also indicate that TEX_{86} may record the temperature change of a different water mass/process from the local surface. In summary, isoGDGT production remains at the depth of the NO_3^- maximum and thus records lower temperatures as the thermocline shoals. Alkenone production remains in the photic zone therefore is unaffected by thermocline shoaling [6,7]. At Site U1475, temperatures deduced from TEX_{86} and $U^{K'}_{37}$ converge at times during the mPWP. However, a recovery in GDGT-0/crenarchaeol and/or the Ring Index, indicating thermocline deepening, does not occur until 3000–2900 ka.

Site 625

IsoGDGTs dominate over branched GDGTs at Site 625 as well. For isoGDGTs, all confounding factor values are well below their cut-off thresholds, except GDGT-2/GDGT-3 (Fig. S5f), which is 5–6, indicating that a substantial component of GDGTs was derived from below the thermocline, similar to at Site U1475.
TEX_{86} values range between 0.54 and 0.67, corresponding to a temperature range of 21–26°C using the TEX_{86}^{H} calibration (Extended Data Fig. 5). Notably, a stepwise 3°C decline in TEX_{86}-based temperature also occurs around 3400 ka, which is synchronous with Site U1475, while $U^{K'}_{37}$ remains relatively stable. Similarly to Site U1475, GDGT-0/crenarchaeol increased with 0.2 and RI decreased with 0.2 units from 3400 ka to MIS M2 (Extended Data Fig. 5). Synchronous with Site U1475, Site 625 exhibits an increase in RI during 3000–2900 ka, which also coincides with thermocline deepening recorded at Site 959 [8]

Site 1087 and 959

The studies of Petrick et al.[9,10] show that $U^{K'}_{37}$- and TEX_{86}- based temperatures are also offset at Site 1087. However, the gradual diverging trend that we see at Sites U1475 and 625 is lacking in this record. We therefore follow the interpretation of the authors that Site 1087 was influenced by upwelling during the studied period. Since only GDGT-based SSTs were reported rather than GDGT peak areas or fractional abundances, compositional changes in GDGT distributions around 3400ka could not be assessed.

Site 959 is located in a region that is currently under the influence of seasonal upwelling, but clear thermocline change during the Pliocene has been indicated[3,8]. Given that the $U^{K'}_{37}$- and TEX_{86}-based temperatures show the same diverging trends as found at Site 625 and Site U1475, we interpret this as evidence for a basin-scale thermocline change across the Atlantic, superimposed on regional upwelling dynamics.

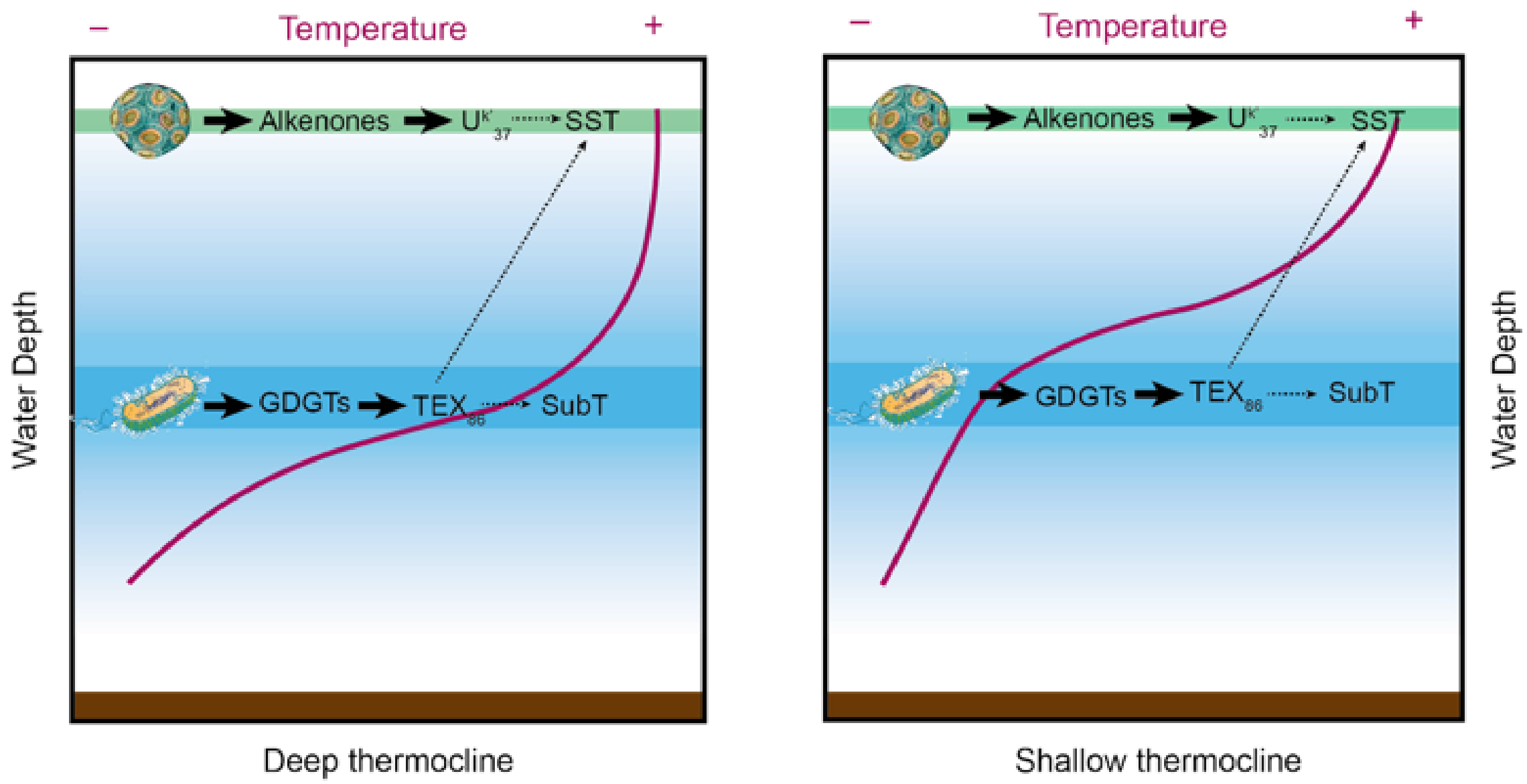

Figure S1. A conceptual sketch of the link between thermocline change and how it is reflected in biomarker data. Shades and gradients indicate the export depth of respective biomarkers; green shade=photic zone, blue shade=NO_3^- rich zone, red line=temperature profile; solid arrows indicate production, dashed arrows indicate to which depth the proxy is calibrated. A thermocline shoaling has the largest effect on TEX_{86}, since the GDGTs on which this index is based are produced and exported predominantly from the depth close to the thermocline. Note that TEX_{86} records that are translated into SSTs will reflect the same trend, as they are presumed to have a constant offset with the subsurface temperatures they actually represent.

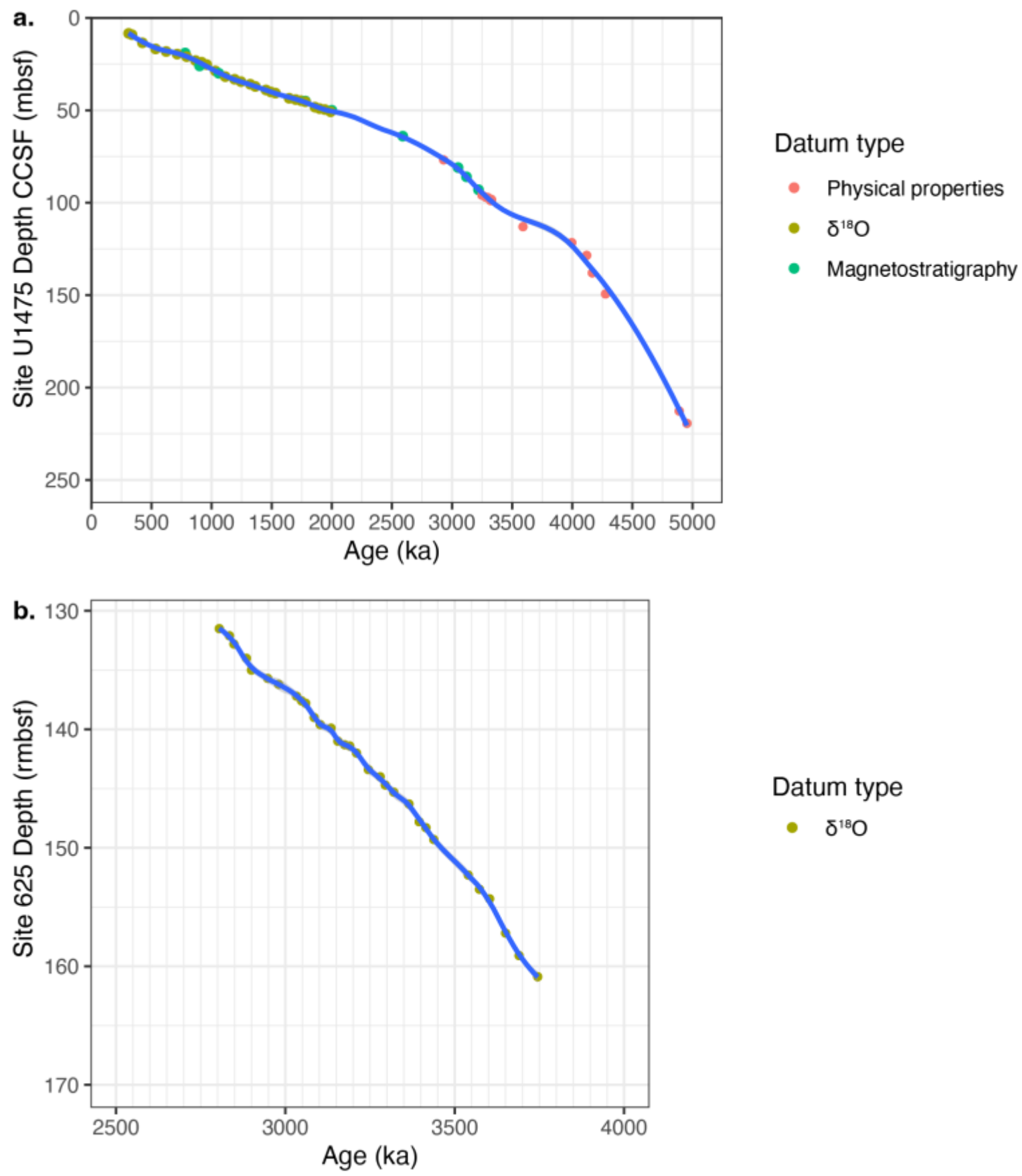

Figure S2. Age models of Site U1475 and Site 625. (a) Site U1475 age model against composite depth replotted based on Starr et al. [11] and Tangunan et al. [12]. **(b)** Site 625 age model against revised depth scale replotted based in Dearing Crampton-Flood et al. [13].

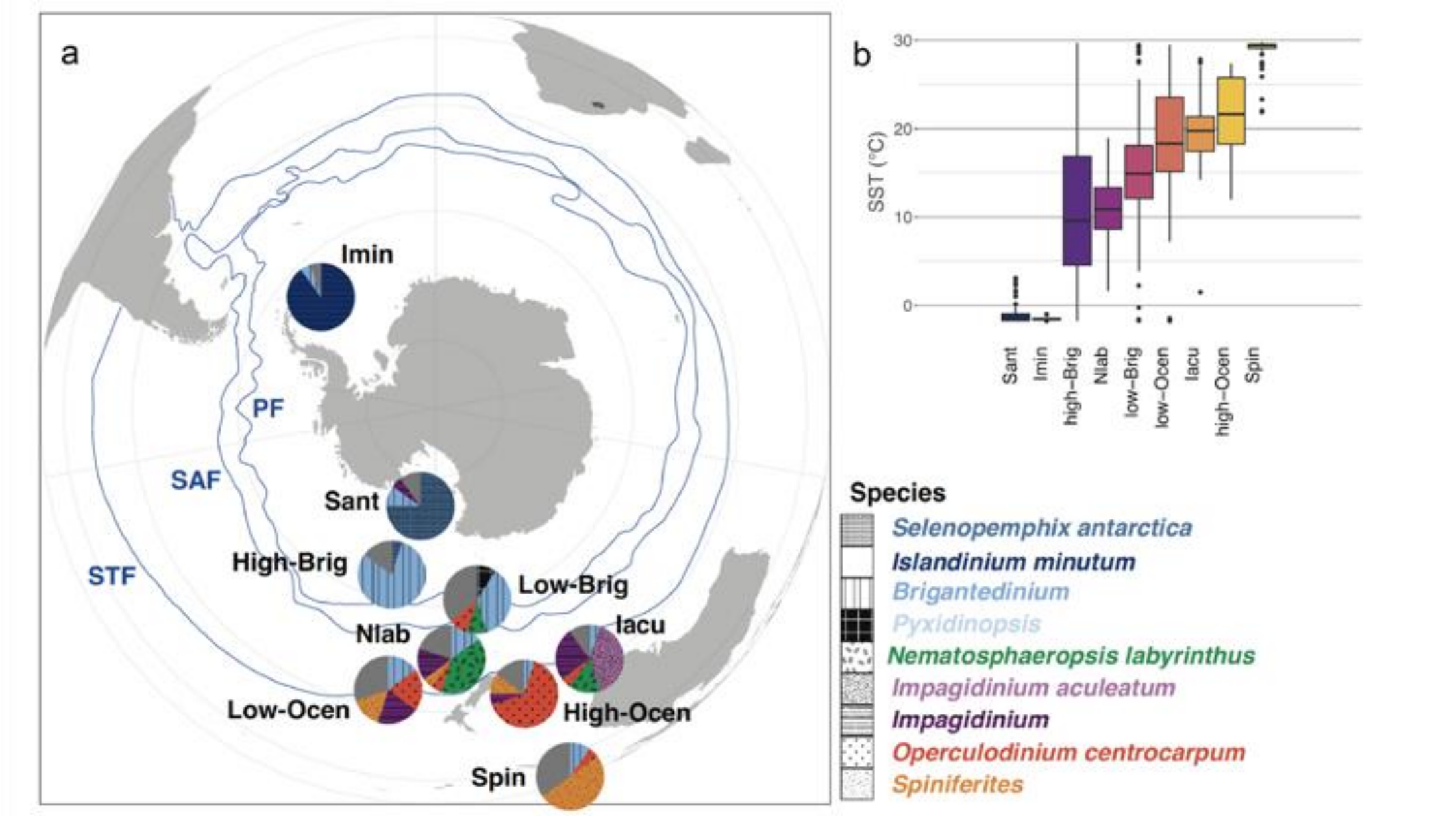

Figure S3. Modern distribution of dinoflagellate cyst in the Southern Ocean. (a) Schematic representation of the generalized biogeographic distribution of dinocysts in Southern Ocean surface sediments. Pies represent average assemblage composition of the nine clusters described in this paper. Position of these pies represent their typical latitudinal band of occurrence. Also plotted are the frontal systems (blue lines, STF = Subtropical Front, SAF = Subantarctic Front, PF = Polar Front). The Subantarctic Zone (SAZ) is the water mass between the STF and PF. **(b)** Comparison of SST in different clusters for the 9-cluster solution of the sh_655 data set. The median, 25% – 75% quantiles and 95% confidence interval are indicated by the black line, boxes and whiskers, respectively. Modified from Thöle et al. [14] under a Creative Commons Attribution 4.0 License.

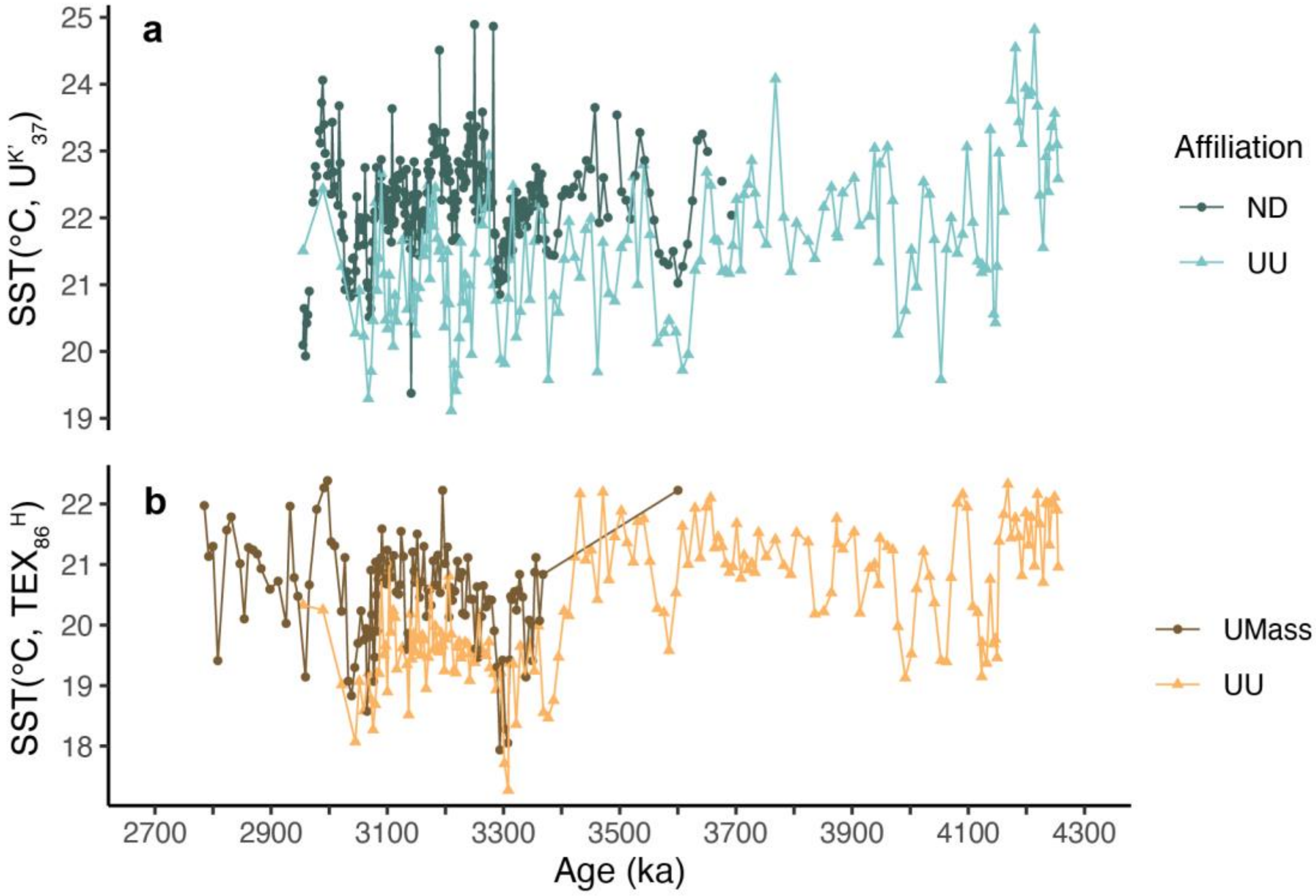

Figure S4. Comparison of lipid biomarker proxy data for Site U1475.SST (a) $U^{K'}_{37}$-derived Sea Surface Temperatures (SSTs) using a linear core-top calibration[15] **(b)** TEX_{86}-derived SSTs using an exponential core-top calibration[4]. UU=Utrecht University, ND= University of Notre Dame, UMass= University of Massachusetts Amherst.

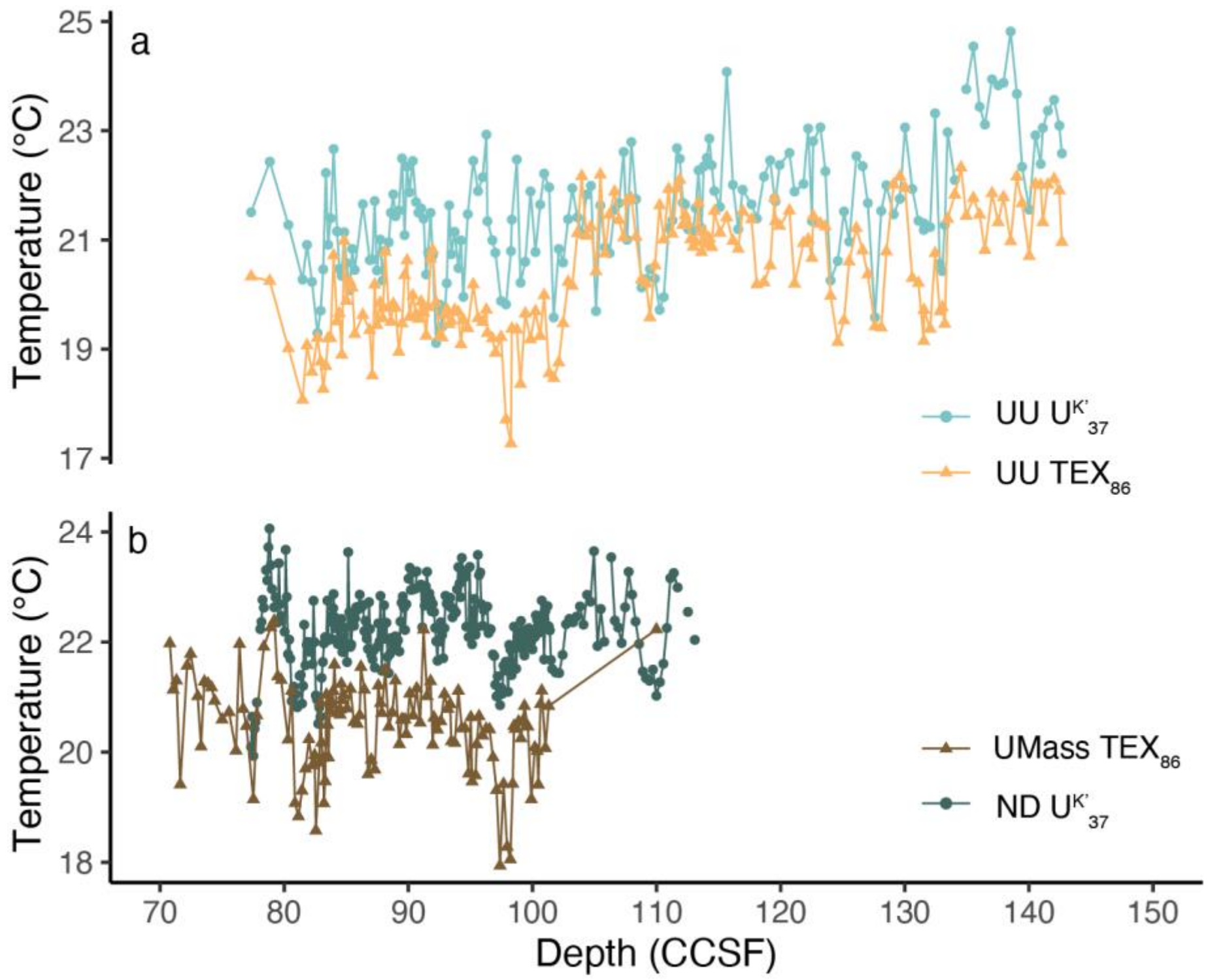

Figure S5. Comparison of lipid biomarker proxy results for Site U1475. (a) $U^{K'}_{37}$ and TEX_{86} -based temperature records generated at Utrecht University (UU). **(b)** $U^{K'}_{37}$ and TEX_{86} -based temperature records generated at the University of Notre Dame (ND) and the University of Massachusetts Amherst (UMass) respectively.

Table S1.

Depth CCSF (m)	Age (ka)	Datum type	Event	Source
13.41	423	$\delta^{18}O$		Starr et al., 2021
15.27	472	$\delta^{18}O$		Starr et al., 2021
16.9	537	$\delta^{18}O$		Starr et al., 2021
18.14	620	$\delta^{18}O$		Starr et al., 2021
19	773	Magnetostratigraphy	b brunhes c1n	Hall et al., 2017
19.54	710	$\delta^{18}O$	MIS 18/17	Starr et al., 2021
20.175	744	$\delta^{18}O$		Starr et al., 2021
21.002	788	$\delta^{18}O$	MIS 20/19	Starr et al., 2021
23.087	866	$\delta^{18}O$	MIS 22/21	Starr et al., 2021
23.94	918	$\delta^{18}O$	MIS 24/23	Starr et al., 2021
25.3	958	$\delta^{18}O$	MIS 26/25	Starr et al., 2021
26	1008	Magnetostratigraphy	b c1r.1r	Hall et al., 2017
26.1	982	$\delta^{18}O$		Starr et al., 2021
28.62	1032	$\delta^{18}O$	MIS 30/29	Starr et al., 2021
29.46	1062	$\delta^{18}O$	MIS 31/30	Starr et al., 2021
30	1076	Magnetostratigraphy	b jaramillo c1r.1n	Hall et al., 2017
30.94	1088	$\delta^{18}O$	MIS 32/31	Starr et al., 2021
31.82	1118	$\delta^{18}O$	MIS 34/33	Starr et al., 2021
33.22	1190	$\delta^{18}O$	MIS 36/35	Starr et al., 2021
34.457	1244	$\delta^{18}O$		Starr et al., 2021
35.855	1320	$\delta^{18}O$		Starr et al., 2021
37.1	1364	$\delta^{18}O$		Starr et al., 2021
37.94	1404	$\delta^{18}O$	MIS 46/45	Starr et al., 2021
38.231	1428	$\delta^{18}O$	MIS 47/46	Starr et al., 2021
38.91	1452	$\delta^{18}O$		Starr et al., 2021
40.08	1494	$\delta^{18}O$	MIS 52/51	Starr et al., 2021
40.72	1530	$\delta^{18}O$	MIS 54/53	Starr et al., 2021
41.8	1570	$\delta^{18}O$	MIS 54/53	Starr et al., 2021
43.32	1642	$\delta^{18}O$		Starr et al., 2021
45	1775	Magnetostratigraphy	b c1r	Hall et al., 2017
50	1934	Magnetostratigraphy	b olduvai c2n	Hall et al., 2017
64	2610	Magnetostratigraphy	b matuyama c2r.2r	Hall et al., 2017
81	3032	Magnetostratigraphy		Hall et al., 2017

86	3120	Magnetostratigraphy		Hall et al., 2017
93	3207	Magnetostratigraphy		Hall et al., 2017
97.5	3290	Physical property stack		Tangunan et al., 2024
113	3590	Physical property stack		Tangunan et al., 2024
121.6	3996	Physical property stack		Tangunan et al., 2024
128.5	4120	Physical property stack		Tangunan et al., 2024
138	4166.5	Physical property stack		Tangunan et al., 2024
149.38	4276.7	Physical property stack		Tangunan et al., 2024
212.76	4889.8	Physical property stack		Tangunan et al., 2024
219.4	4954	Physical property stack		Tangunan et al., 2024

Table S1: Age model tie-points of Site U1475[11,12,16].

Table S2.

Sample ID	Affiliation	TEX_{86}	TEX_{86} difference
1475B 13H-5W 138-140	UMass	0.5870	
1475B-13H-5W 138-140	UU	0.5750	0.0120
1475B 9H-4W 45-48	UMass	0.5473	
1475B 9H-4W-45-48	UU	0.5182	0.0292
1475E 13H-6W 88-90	UMass	0.5529	
1475E 13H-6W 88-90	UU	0.5296	0.0233
1475B 14H-1W 138-140	UMass	0.6025	
1475B 14H-1W 138-140	UU	0.5782	0.0243
1475B 14H-3W 138-140	UMass	0.5843	
1475B 14H-3W 138-140	UU	0.5590	0.0253
1475B 9H-6W 63-65	UMass	0.5604	
1475B 9H-6W 63-65	UU	0.5326	0.0278
1475B 10H-2W 90-92	UMass	0.5570	
1475B 10H-2W 90-92	UU	0.5161	0.0409
1475B 10H-3W 138-140	UMass	0.5614	
1475B 10H-3W 138-140	UU	0.5286	0.0328
1475B 10H-6W 10-12	UMass	0.5439	
1475B 10H-6W 10-12	UU	0.5263	0.0175
1475B 11H-6W 67-69	UMass	0.5781	
1475B 11H-6W 67-69	UU	0.5616	0.0164
1475B 12H-2W 10-12	UMass	0.5942	
1475B 12H-2W 10-12	UU	0.5738	0.0204
1475F 7H-3W 127-129	UMass	0.5729	
1475F 7H-3W 127-129	UU	0.5673	0.0056
1475B 9H-4W 45-48	UMass	0.5473	
1475B 9H-4W 45-48	UU	0.5182	0.0292
1475B 10H-7W 45-48	UMass	0.5444	
1475B 10H-7W 45-48	UU	0.5297	0.0147
1475B 12H-3W 34-37	UMass	0.5775	
1475B 12H-3W 34-37	UU	0.5488	0.0287
1475B 13H-6W 30-33	UMass	0.5892	
1475B 13H-6W 30-33	UU	0.5709	0.0182
median			0.0238
average			0.0229
SD			0.0085

Table S2: Interlaboratory comparison of TEX_{86} results between Utrecht University (UU) and University of Massachusetts Amherst (UMass).

Table S3.

Sample ID	$U^{K'}_{37}$-ND	$U^{K'}_{37}$-UU	$U^{K'}_{37}$ difference
1475B 13H5W 139K	0.7443	0.7340	0.0104
1475E 13H6W 89K	0.7442	0.7470	-0.0028
1475B 14H3W 139K	0.8261	0.8305	-0.0044
1475B 14H6W 89K	0.8071	0.8151	-0.0080
1475B 9H6W 64K	0.7221	0.7230	-0.0009
1475B 10H2W 91K	0.7515	0.7548	-0.0033
1475B 10H3W 139K	0.7598	0.7498	0.0100
1475B 10H6W 11K	0.7095	0.7026	0.0069
1475B 11H6W 68K	0.7426	0.7289	0.0137
1475B 12H2W 11K	0.7931	0.7861	0.0070
1475F 7H3W 128K	0.7588	0.7623	-0.0035
1475B 9H4W 45-48K	0.7366	0.7340	0.0026
1475B 10H7W 45-48K	0.7980	0.8007	-0.0027
1475B 12H3W 34-37K	0.7563	0.7442	0.0121
1475B 13H6W 30-33K	0.8045	0.8050	-0.0004
ND STD F2	0.7943	0.7845	0.0098
ND STD F2	0.7901	0.7870	0.0031
ND STD F2	0.7888	0.7940	-0.0052
ND STD F2	0.7951	0.7934	0.0017
ND STD F2	0.7845	0.7908	-0.0063
ND STD F2	0.7882	0.7881	0.0001
median			-0.0004
average			0.0024
SD			0.0067

Table S3: Interlaboratory comparison of $U^{K'}_{37}$ results between Utrecht University (UU) and University of Notre Dame (ND).

References

1. Hurley, S. J., Lipp, J. S., Close, H. G., Hinrichs, K.-U. & Pearson, A. Distribution and export of isoprenoid tetraether lipids in suspended particulate matter from the water column of the Western Atlantic Ocean. *Organic Geochemistry* **116**, 90–102 (2018).
2. Taylor, K. W. R., Huber, M., Hollis, C. J., Hernandez-Sanchez, M. T. & Pancost, R. D. Re-evaluating modern and Palaeogene GDGT distributions: Implications for SST reconstructions. *Global and Planetary Change* **108**, 158–174 (2013).
3. Van der Weijst, C. M. H. *et al.* A 15-million-year surface- and subsurface-integrated TEX_{86} temperature record from the eastern equatorial Atlantic. *Climate of the Past* **18**, 1947–1962 (2022).
4. Kim, J.-H. *et al.* New indices and calibrations derived from the distribution of crenarchaeal isoprenoid tetraether lipids: Implications for past sea surface temperature reconstructions. *Geochimica et Cosmochimica Acta* **74**, 4639–4654 (2010).
5. Zhang, Y. G., Pagani, M. & Wang, Z. Ring Index: A new strategy to evaluate the integrity of TEX_{86} paleothermometry. *Paleoceanography* **31**, 220–232 (2016).
6. Lopes dos Santos, R. A. *et al.* Glacial–interglacial variability in Atlantic meridional overturning circulation and thermocline adjustments in the tropical North Atlantic. *Earth and Planetary Science Letters* **300**, 407–414 (2010).
7. Sagawa, T., Yokoyama, Y., Ikehara, M. & Kuwae, M. Shoaling of the western equatorial Pacific thermocline during the last glacial maximum inferred from multispecies temperature reconstruction of planktonic foraminifera. *Palaeogeography, Palaeoclimatology, Palaeoecology* **346–347**, 120–129 (2012).
8. Van der Weijst, C. M. H. *et al.* Pliocene evolution of the tropical Atlantic thermocline depth. *Climate of the Past* **18**, 961–973 (2022).
9. Petrick, B. *et al.* Late Pliocene upwelling in the Southern Benguela region. *Palaeogeography, Palaeoclimatology, Palaeoecology* **429**, 62–71 (2015).
10. Petrick, B. *et al.* Oceanographic and climatic evolution of the southeastern subtropical Atlantic over the last 3.5 Ma. *Earth and Planetary Science Letters* **492**, 12–21 (2018).
11. Starr, A. *et al.* Antarctic icebergs reorganize ocean circulation during Pleistocene glacials. *Nature* **589**, 236–241 (2021).
12. Tangunan, D. N. *et al.* Southern Ocean and Southern African Monsoon Forcing of the Subtropical Indian Ocean Early Pliocene "Biogenic Bloom". *Paleoceanography and Paleoclimatology* **39**, e2024PA004927 (2024).
13. Dearing Crampton-Flood, E. *et al.* Identifying marine and freshwater overprints on soil-derived branched GDGT temperature signals in Pliocene Mississippi and Amazon River fan sediments. *Organic Geochemistry* **154**, 104200 (2021).
14. Thöle, L. M. *et al.* An expanded database of Southern Hemisphere surface sediment dinoflagellate cyst assemblages and their oceanographic affinities. *Journal of Micropalaeontology* **42**, 35–56 (2023).
15. Müller, P. J., Kirst, G., Ruhland, G., von Storch, I. & Rosell-Melé, A. Calibration of the alkenone paleotemperature index U37K′ based on core-tops from the eastern South Atlantic and the global ocean (60°N-60°S). *Geochimica et Cosmochimica Acta* **62**, 1757–1772 (1998).
16. Hall, I. R. *et al.* IODP Publications • Volume 361 expedition reports • Site U1475. https://doi.org/doi.org/10.14379/iodp.proc.361.104.2017 (2017) doi:doi.org/10.14379/iodp.proc.361.104.2017.
