# Supplementary material for: Disconnection of the late Pliocene Agulhas Leakage from Atlantic Meridional Overturning Circulation
Source: Nat Geosci. 2026 Aug 3;19(10):1158–66. doi: 10.1038/s41561-026-02055-5 (PMC13645622; doi:10.1038/s41561-026-02055-5)
Supplement: Supplementary file 2 [file 41561_2026_2055_MOESM2_ESM.pdf]

nature portfolio

Peer Review File

Disconnection of the Late Pliocene Agulhas Leakage to Atlantic Meridional Overturning

Corresponding Author: Dr Suning Hou

Version 0:

Reviewer comments:

Reviewer #1

(Remarks to the Author)
To whom it may concern:
I was asked to review the following article Agulhas Leakage decoupled from Atlantic Meridional Overturning in the Late Pliocene." After reading the article, I think this is not only publishable but that the findings are significant. However, I believe there is one major issue that needs to be corrected. As some of this relates to my research, I will be publicly reviewing this article to ensure the authors do not feel I am being underhanded.

The way that the article is written suggests that the location STF controls the Agulhas Leakage (Stroger further south, weaker further north), and therefore, they can test the Agulhas leakage's connections with AMOC. However, the theory that STF location played any role in the strength of the recent glacial and interglacial Agulhas Leakage is highly debated. In fact, given that the increase in the Agulhas Leakage only occurs during deglaciations, it suggests that the relationship was very complex, as seen in modern records (Caley et al., 2012, 2014; B. F. Petrick et al., 2015; van Sebille et al., 2009) and my previous work in the Pliocene in the "leakage region" showed no evidence of leakage (B. Petrick et al., 2015; Petrick et al., 2018). Therefore, I think the authors have missed a crucial step in first showing that the STF influences Agulhas leakage. However, since the missing component in all these studies is the lack of data on the location of the STF, the authors possess a critically essential data set. Therefore, the finding that even with some shifts in area, the STF plays NO role in the apparent lack of Agulhas leakage in the Pliocene is significant not only because it shows that the Agulhas leakage doesn't fit changes in AMOC, but more importantly, it shows changes in the STF do not seem to lead to changes in the Agulhas leakage system. It also fits with my previous findings from ODP site 1087, which showed no evidence of Agulhas leakage (which the authors need to acknowledge the findings of more in the paper, especially as they do not have a "leakage" site of their own, which makes 1087 the only "leakage" site with a Pliocene record). Therefore, I suggest that the authors refocus the article on the debate about the effect of the STF on the Agulhas and how their new data provides a new perspective on that debate, as well as the wider AMOC system. The good news is that this is all restructuring and expansion, and that can be done with minor corrections, so this is what I suggest.

Line 38: Please expand this. There has been some debate on whether the STF truly controls the Agulhas Leakage and if there is a one-to-one connection. More needs to be said here.
Line 61-62: Again, what is this based on? The only citation is Beal et al. (2011). I know more has been done on this question since then.

Line 92-94: isn't this unexpected cooling of the TEX86 upwelling see (B. Petrick et al., 2015; Petrick et al., 2018). Also, looking at the figure in the supplement, the difference is only in the BAYSPAR and not the TEX86H. More needs to be here or in the supplement on why you decided to trust the BAYSPAR over TEX86H

Line 101-103: Bard and Rickaby (2009) have been debated for a while, and it has been thought that they exaggerated the northward movement of the STF. I would like to see some of the more recent work on this, quoted here.

Line 123-134: ODP site 1087 also shows an offset between TEX86 and UK37' during the Pliocene. See Petrick et al (2015 and 2018). I am wondering, given that almost all of the sites along the transect show this, whether this is structural or just changes in upwelling intensity.

Line 177: I am not sure that I would necessarily assume that further northward STF equals more substantial Agulhas leakage, see my suggestion above

Line 222-226: This is really interesting because this suggests that the Agulhas Leakage operated during M2, like in the

modern, with a spike on deglaciation. Given this is the only clear Agulhas Leakage signal seen in the Pliocene in this record, I see the possibility here for a better understanding between Agulhas Leakage and STF migration. This should be expanded.

Line 252: see Petrick et al (2015). Also, it is not as established in modern studies as suggested here.

Line 470-494: The offset between the two laboratories is concerning. I would first like to see a figure showing the offset between SSTs in the same proxy. While they plot (in the supplement) the raw data for TEX86 and UK37' on the same axis, they plot the two temperatures by laboratory. I would also like to have some discussion about this, at least in the supplement. Having completed several projects involving multiple labs, I have not often seen this kind of offset. While I don't think it should affect the conclusions of the paper or invalidate them, I feel this is currently a little buried. I would feel more confident in the data with a discussion. Given the occasional skepticism about biomarker proxies, we need to do this kind of work. Again, I recommend Minor Corrections.

Benjamin Petrick

Citations
Caley, T., Giraudeau, J., Malaize, B., Rossignol, L., Pierre, C., 2012. Agulhas leakage as a key process in the modes of Quaternary climate changes. Proceedings of the National Academy of Sciences 109, 6835–6839. https://doi.org/10.1073/pnas.1115545109
Caley, T., Peeters, F.J.C., Biastoch, A., Rossignol, L., Van Sebille, E., Durgadoo, J., Malaizé, B., Giraudeau, J., Arthur, K., Zahn, R., 2014. Quantitative estimate of the paleo-Agulhas leakage. Geophys Res Lett 41, 1238–1246. https://doi.org/10.1002/2014GL059278
Petrick, B., McClymont, E.L., Felder, S., Rueda, G., Leng, M.J., Rosell-Melé, A., 2015. Late Pliocene upwelling in the Southern Benguela region. Palaeogeogr Palaeoclimatol Palaeoecol 429, 62–71. https://doi.org/10.1016/j.palaeo.2015.03.042
Petrick, B., McClymont, E.L., Littler, K., Rosell-Melé, A., Clarkson, M.O., Maslin, M., Röhl, U., Shevenell, A.E., Pancost, R.D., 2018. Oceanographic and climatic evolution of the southeastern subtropical Atlantic over the last 3.5 Ma. Earth Planet Sci Lett 492, 12–21. https://doi.org/10.1016/j.epsl.2018.03.054
Petrick, B.F., McClymont, E.L., Marret, F., van der Meer, M.T.J., 2015. Changing surface water conditions for the last 500 ka in the Southeast Atlantic: Implications for variable influences of Agulhas leakage and Benguela upwelling. Paleoceanography 30, 1153–1167. https://doi.org/10.1002/2015PA002787
van Sebille, E., Biastoch, A., van Leeuwen, P.J., de Ruijter, W.P.M., 2009. A weaker Agulhas Current leads to more Agulhas leakage. Geophys. Res. Lett. 36. https://doi.org/10.1029/2008gl036614

Reviewer #2

(Remarks to the Author)
Hou et al. reconstruct the position of the Southern Ocean subtropical front (STF) using dinoflagellate cysts and biomarkers from Agulhas Plateau, which they interpret as a proxy for Agulhas Leakage (AL). Based on their reconstruction, they argue for a weakening of AL from 3600 to 3300ka, during a period when AMOC is thought to have strengthened. From this, they infer a decoupling between AL and AMOC. However, several uncertainties weaken this logical chain and limit the strength of their conclusion.

First, the inferred reduction in AL is derived solely from changes in the STF position. Yet AL variability is also strongly modulated by surface wind forcing. Without independent constraints on wind strength or additional, more direct AL proxies, the interpretation of STF migration as an indicator of AL variability remains equivocal.

Second, AMOC strength is influenced by multiple processes, most prominently North Atlantic surface buoyancy forcing and Southern Ocean wind stress (e.g., Toggweiler & Samuels, 1995). Variations in these drivers could offset or overwhelm any contribution from AL. Without evaluating concurrent changes in these other forcings, the argument that AL and AMOC were decoupled during this interval is not sufficiently supported.

Minor:
Line 41-44: Please briefly describe the relevant debates and uncertainties, as the current wording is too vague. Readers who are not familiar with the cited literature may find the statements unclear. For example, what kinds of debate in Line 41, whether or not AL can influence AMOC under modern condition in Line 43? Providing a brief explanation of these differing viewpoints would make your argument much clearer to readers.

Line 57: It is unclear what the "critical knowledge gap" refers to in the current introduction. From the contents above, it is hard to understand what is the current gap. The overall motivation for this study is not fully established.

Line 123-128: why the increase Uk37 and TEX86 gradient indicates the thermocline shoaling? Is this a well-established phenomenon in the previous publications and what is the mechanism behind this relationship.

Line 164-165: It is hard to reconcile the stated “weaken AMOC upper limb” and “increased overturning” at the same time because the upper limb and the lower limb should change in-phase as the lower limb is the return flow of the AMOC. The core of the AMOC upper cell locates at ~750m. If the AMOC at MIS M2 is with similar geometry as in mPWP, it seems the AMOC upper cell is weaker from Figure 4b. The AMOC at MIS M2 should be provided in addition to the anomaly plot. The strength of AMOC is usually defined as the maximum overturning streamfunction in the North Atlantic at mid-depth. The specific values of AMOC strength at mPVP and MIS M2 should be provided.

Line 180-181: Why the decrease of the temperature gradient between 40N and 53N infers a decrease suppler of NAC? NAC transports heat to the high latitude, therefore stronger NAC should lead to weaker SST gradient.

Line 182-183: The salinity change in the Caribbean Sea could also be affected by AAIW.

Line 204: It is more appropriate to identify the NADW formation region from the winter mixed layer depth.

Line 207: What is the dynamical mechanism between thermocline shoaling and NADW intensification?

Reference:
Toggweiler, J. R., & Samuels, B. (1995). Effect of drake passage on the global thermohaline circulation. Deep-Sea Research Part I, 42(4), 477–500. https://doi.org/10.1016/0967-0637(95)00012-U

Version 1:

Reviewer comments:

Reviewer #1

(Remarks to the Author)
I was asked to re-review the article “Agulhas Leakage disconnected to Atlantic Meridional Overturning in the Late Pliocene”. I would like to start by thanking the authors for their response to the reviewers and for their very organized approach to presenting the corrections. I also appreciate that, in the rare cases when they disagree with my suggestions, they explain their reasoning thoughtfully. I find the organization really brings out the major point, which I think makes this article hugely significant: the connections between the AL, STF, and AMOC are much more complex than initial studies have suggested. I recommend strengthening the final statement and making it clear that this may not hold for the 100 ka world, as several studies have suggested. However, I will leave that up to the authors. I have one note:
Age Models: Can you add a table with tie points for the new adjusted age model or provide a link to where such a table can be found? From experience, this will help future researchers who work on this core.
I think you can send this directly to the editor during the proofs stage.
Thanks again for your hard work and congratulations on a significant study.
Dr. Benjamin Petrick

Reviewer #2

(Remarks to the Author)
I thank the authors for carefully addressing my previous comments, particularly their inclusion of wind forcing effects on Agulhas Leakage and the added discussion on the driving mechanisms of the AMOC. These revisions have improved the clarity and completeness of the manuscript. I have no further questions or concerns.

Open Access This Peer Review File is licensed under a Creative Commons Attribution 4.0 International License, which permits use, sharing, adaptation, distribution and reproduction in any medium or format, as long as you give appropriate credit to the original author(s) and the source, provide a link to the Creative Commons license, and indicate if changes were made.
In cases where reviewers are anonymous, credit should be given to 'Anonymous Referee' and the source.
The images or other third party material in this Peer Review File are included in the article's Creative Commons license, unless indicated otherwise in a credit line to the material. If material is not included in the article's Creative Commons license and your intended use is not permitted by statutory regulation or exceeds the permitted use, you will need to obtain permission directly from the copyright holder.
To view a copy of this license, visit https://creativecommons.org/licenses/by/4.0/

Reviewers’ comments:

Reviewer #1 (Remarks to the Author):

To whom it may concern:
I was asked to review the following article Agulhas Leakage decoupled from Atlantic Meridional Overturning in the Late Pliocene.” After reading the article, I think this is not only publishable but that the findings are significant. However, I believe there is one major issue that needs to be corrected. As some of this relates to my research, I will be publicly reviewing this article to ensure the authors do not feel I am being underhanded.
Reply: We thank the reviewer for the interest and constructive comments.

The way that the article is written suggests that the location STF controls the Agulhas Leakage (Stroger further south, weaker further north), and therefore, they can test the Agulhas leakage’ s connections with AMOC. However, the theory that STF location played any role in the strength of the recent glacial and interglacial Agulhas Leakage is highly debated. In fact, given that the increase in the Agulhas Leakage only occurs during deglaciations, it suggests that the relationship was very complex, as seen in modern records (Caley et al., 2012, 2014; B. F. Petrick et al., 2015; van Sebille et al., 2009) and my previous work in the Pliocene in the “leakage region” showed no evidence of leakage (B. Petrick et al., 2015; Petrick et al., 2018). Therefore, I think the authors have missed a crucial step in first showing that the STF influences Agulhas leakage. However, since the missing component in all these studies is the lack of data on the location of the STF, the authors possess a critically essential data set. Therefore, the finding that even with some shifts in area, the STF plays NO role in the apparent lack of Agulhas leakage in the Pliocene is significant not only because it shows that the Agulhas leakage doesn’ t fit changes in AMOC, but more importantly, it shows changes in the STF do not seem to lead to changes in the Agulhas leakage system. It also fits with my previous findings from ODP site 1087, which showed no evidence of Agulhas leakage (which the authors need to acknowledge the findings of more in the paper, especially as they do not have a “leakage” site of their own, which makes 1087 the only “leakage” site with a Pliocene record). Therefore, I suggest that the authors refocus the article on the debate about the effect of the STF on the Agulhas and how their new data provides a new perspective on that debate, as well as the wider AMOC system. The good news is that this is all restructuring and expansion, and that can be done with minor corrections, so this is what I suggest.
Reply: We agree with the reviewer and thank the reviewer for pointing out the importance of the articles.
The reviewer argues whether there was any change in AL in the Pliocene despite vigorous frontal shifts. In our opinion there can be two scenarios: (1) STF migration did not cause any changes in the AL, as found at Site 1087 (Petrick et al., 2015; 2018). Or (2) STF caused the changes in the AL (as proposed in our original manuscript)

which were not captured by Site 1087 (note that this possibility was also mentioned in Petrick et al. 2015; 2018). In both scenarios, however, there is no relationship between AL and AMOC strength, which is a hypothesis that is still followed by many researchers studying Agulhas Leakage.

Correction: We have included reviewer' s opinion in the introduction and discussion. We better clarify the link between Southern Ocean wind stress, STF position and AL in the introduction meanwhile acknowledging the complexity. We adjust the discussion by highlighting the two scenarios mentioned above, explicitly expand the discussion regarding Site 1087 biomarker records and incorporate the dust record (XRF, Ti/Al) into Figure 2, as well as the fact that model simulations do show STF shifts and AL changes. For details, please refer to the revised manuscript lines 37-42, 125-137.

Line 38: Please expand this. There has been some debate on whether the STF truly controls the Agulhas Leakage and if there is a one-to-one connection. More needs to be said here.

Correction: We agree with the reviewer and have expanded accordingly, as also requested by Reviewer2. Please refer to lines 37-42, 62-64.

Line 61-62: Again, what is this based on? The only citation is Beal et al. (2011). I know more has been done on this question since then.

Correction: We have added references (e.g., Caley et al., 2012) (lines 79-80) and expanded the introduction in general as mentioned above.

Line 92-94: isn' t this unexpected cooling of the TEX86 upwelling see (B. Petrick et al., 2015; Petrick et al., 2018). Also, looking at the figure in the supplement, the difference is only in the BAYSPAR and not the TEX86H. More needs to be here or in the supplement on why you decided to trust the BAYSPAR over TEX86H

Reply: We thank the reviewer for this suggestion. Both BAYSPAR and TEX86H show a divergence from UK' 37 from 3400ka onwards, but this divergence is more prominent in the BAYSPAR record. Extended data figure 3 shows that there is an abrupt change in GDGT composition at 3400ka. This is reflected in the Ring Index and GDGT-0/crenarchaeol, as well as in the TEX86, The lower TEX86-SSTs thus represent the lower temperature of the subsurface. In addition, our dinoflagellate cyst record shows no signs of upwelling, an oceanographic change that dinocysts are actually very responsive to..

Correction: The information above can be found in the supplementary discussion part. We also expand the supplementary discussion by adding Site 1087 and 959.

Line 101-103: Bard and Rickaby (2009) have been debated for a while, and it has been thought that they exaggerated the northward movement of the STF. I would like to see some of the more recent work on this, quoted here.

Reply: We thank the reviewer for providing some community comments on this reference.

Correction: We have toned down this statement and included additional references (Lu et al., 2025; Starr et al., 2025; Wu et al., 2026). Please refer to lines 110-114

Line 123-134: ODP site 1087 also shows an offset between TEX86 and UK37' during the Pliocene. See Petrick et al (2015 and 2018). I am wondering, given that almost all of the sites along the transect show this, whether this is structural or just changes in upwelling intensity.

Reply: Sites 1087 and 959 are indeed located in upwelling areas, but Sites U1475 and 625 are not. More importantly, what we observed is the gradual divergence between TEX86- and UK'37-SSTs. Site 1087 seems to have a consistent difference, maybe also due to the resolution of TEX86. However, previous studies on Site 959 indicate a clear change in the thermocline depth (Van der Weijst et al., 2022b, a).

Correction: We have expanded this in the supplementary discussion as mentioned further above.

Line 177: I am not sure that I would necessarily assume that further northward STF equals more substantial Agulhas leakage, see my suggestion above

Reply: We are afraid the reviewer is mistake here. We have linked a northward position of STF to less Agulhas Leakage. Now we focus more on Petrick et al., (2015, 2018) papers and address the reviewer's major concern as described above.

Line 222-226: This is really interesting because this suggests that the Agulhas Leakage operated during M2, like in the modern, with a spike on deglaciation. Given this is the only clear Agulhas Leakage signal seen in the Pliocene in this record, I see the possibility here for a better understanding between Agulhas Leakage and STF migration. This should be expanded.

Correction: We expand the discussion here, please refer to lines 254-260.

Line 252: see Petrick et al (2015). Also, it is not as established in modern studies as suggested here.

Reply: We refer to the replies and revisions to the major concern, further above.

Line 470-494: The offset between the two laboratories is concerning. I would first like to see a figure showing the offset between SSTs in the same proxy. While they plot (in the supplement) the raw data for TEX86 and UK37' on the same axis, they plot the two temperatures by laboratory. I would also like to have some discussion about this, at least in the supplement. Having completed several projects involving multiple labs, I have not often seen this kind of offset. While I don't think it should affect the conclusions of the paper or invalidate them, I feel this is currently a little buried. I would feel more confident in the data with a discussion. Given the occasional skepticism about biomarker proxies, we need to do this kind of work.

Reply: We agree with the reviewer that the interlaboratory offset deserves more attention. Firstly, Interlaboratory offsets between datasets are unfortunately quite common. Instrumental settings, choice of columns, even lab conditions affect the details of the final results (Bijl et al., 2025). There are only a few round-robin

interlaboratory comparisons of GDGTs analysis. The largest interlaboratory offset can be up to 0.2 TEX86 unit (Schouten et al., 2013). Thus, the offset (0.03) shown in our results is not unexpected and we have reported it honestly. Consequently, comparisons between records generated by different laboratories should be interpreted with caution and so as model-paleo data comparison, especially when the difference is subtle. Secondly, we note the samples measured in the different laboratories were from different holes and cores at Site U1475. Finally, we note that the differences between laboratories are minor and do not affect the overall interpretation of these records.

Correction: We have changed the y-axis in Figure S3 from proxy unit to temperature as suggested. We have also incorporated the content above briefly into the method part. (see Methods line 411-416).

Again, I recommend Minor Corrections.

Reply: We thank the reviewer Dr. Petrick again for the constructive suggestions that significantly strengthened the manuscript.

Benjamin Petrick

Citations

Caley, T., Giraudeau, J., Malaize, B., Rossignol, L., Pierre, C., 2012. Agulhas leakage as a key process in the modes of Quaternary climate changes. Proceedings of the National Academy of Sciences 109, 6835 - 6839.

Caley, T., Peeters, F.J.C., Biastoch, A., Rossignol, L., Van Sebille, E., Durgadoo, J., Malaizé, B., Giraudeau, J., Arthur, K., Zahn, R., 2014. Quantitative estimate of the paleo-Agulhas leakage. Geophys Res Lett 41, 1238 - 1246.

Petrick, B., McClymont, E.L., Felder, S., Rueda, G., Leng, M.J., Rosell-Melé, A., 2015. Late Pliocene upwelling in the Southern Benguela region. Palaeogeogr Palaeoclimatol Palaeoecol 429, 62 - 71. https://doi.org/10.1016/j.palaeo.2015.03.042

Petrick, B., McClymont, E.L., Littler, K., Rosell-Melé, A., Clarkson, M.O., Maslin, M., Röhl, U., Shevenell, A.E., Pancost, R.D., 2018. Oceanographic and climatic evolution of the southeastern subtropical Atlantic over the last 3.5 Ma. Earth Planet Sci Lett 492, 12 - 21. https://doi.org/10.1016/j.epsl.2018.03.054

Petrick, B.F., McClymont, E.L., Marret, F., van der Meer, M.T.J., 2015. Changing surface water conditions for the last 500 ka in the Southeast Atlantic: Implications for variable influences of Agulhas leakage and Benguela upwelling. Paleoceanography 30, 1153 - 1167. https://doi.org/10.1002/2015PA002787

van Sebille, E., Biastoch, A., van Leeuwen, P.J., de Ruijter, W.P.M., 2009. A weaker Agulhas Current leads to more Agulhas leakage. Geophys. Res. Lett. 36. https://doi.org/10.1029/2008gl036614

Reviewer #2 (Remarks to the Author):

Hou et al. reconstruct the position of the Southern Ocean subtropical front (STF) using dinoflagellate cysts and biomarkers from Agulhas Plateau, which they interpret as a proxy for Agulhas Leakage (AL). Based on their reconstruction, they argue for a weakening of AL from 3600 to 3300ka, during a period when AMOC is thought to have strengthened. From this, they infer a decoupling between AL and AMOC. However, several uncertainties weaken this logical chain and limit the strength of their conclusion.

Reply: We thank the reviewer for their interest and valuable comments from a physical oceanography perspective.

First, the inferred reduction in AL is derived solely from changes in the STF position. Yet AL variability is also strongly modulated by surface wind forcing. Without independent constraints on wind strength or additional, more direct AL proxies, the interpretation of STF migration as an indicator of AL variability remains equivocal.

Reply: We agree with the reviewer and thank for emphasizing the role of wind forcing in AL. Intensified Southern Hemisphere westerlies promote a southward shift of the Antarctic Circumpolar Current (ACC), displacing the subtropical front (STF) poleward and facilitating increased leakage of Indian Ocean water into the Atlantic. Thus, the reviewer’s concern and our interpretation are actually consistent.

Correction: We have clarified the link between Southern Ocean wind stress, STF and AL as above in the introduction. We have also incorporated modelling outputs of wind stress and paleo dust input record of the studied area into the discussion, which is potential indicative of wind field position (Petrick et al., 2018). Please refer to lines 37-42, 125-137.

Second, AMOC strength is influenced by multiple processes, most prominently North Atlantic surface buoyancy forcing and Southern Ocean wind stress (e.g., Toggweiler & Samuels, 1995). Variations in these drivers could offset or overwhelm any contribution from AL. Without evaluating concurrent changes in these other forcings, the argument that AL and AMOC were decoupled during this interval is not sufficiently supported.

Reply: We agree with the reviewer that other processes play important roles. The purpose of our manuscript, however, is to specifically reconcile the effect of AL on AMOC strength proposed by some, with the local oceanographic forcing in the North Atlantic on AMOC proposed by others. Regarding Southern Ocean wind stress through Ekman transport (Toggweiler and Samuels, 1995), based on our understanding from more recent studies, this process seems to have very limited influence on AMOC (e.g., Schulzki et al., 2024). North Atlantic buoyancy forcing is indeed a prominent process. However, (paleo) studies on North Atlantic buoyancy forcing usually ignore the role of AL and vice versa. Our data of the late Pliocene reconcile the two lines of reasoning and assess the viability of interhemispheric teleconnection.

Correction: We incorporate discussions regarding concurrent Greenland meltwater and freshwater input through Arctic gateways into the manuscript. We would like to mention that this part of discussion was considered but was removed before the first submission to reduce the length of our manuscript. Please refer to lines 278-288.

Minor:

Line 41-44: Please briefly describe the relevant debates and uncertainties, as the current wording is too vague. Readers who are not familiar with the cited literature may find the statements unclear. For example, what kinds of debate in Line 41, whether or not AL can influence AMOC under modern condition in Line 43? Providing a brief explanation of these differing viewpoints would make your argument much clearer to readers.

Correction: We adjust the order of the statements and provide more background accordingly. Please refer to lines 46-50, 60-61, .

Line 57: It is unclear what the "critical knowledge gap" refers to in the current introduction. From the contents above, it is hard to understand what is the current gap. The overall motivation for this study is not fully established.

Reply: We mean that Pliocene reconstructions of STF migration and AL are scarce and a combined examination of NAC, NADW and AL is yet missing. This is the knowledge gap we are referring to.

Correction: We have adjusted the order of the statements to make it clear. Please refer to lines 60-66.

Line 123-128: why the increase Uk37 and TEX86 gradient indicates the thermocline shoaling? Is this a well-established phenomenon in the previous publications and what is the mechanism behind this relationship.

Reply: This phenomenon has firstly been described in Lopes dos Santos et al. (2010), which is cited in the manuscript. A more detailed discussion explaining the principles underlaying the Uk37-TEX86 gradient can be found in the supplementary file. We can provide a schematic diagram here in the rebuttal to alleviate this concern.

Correction: We have added the figure below into the supplementary file, which is a conceptual sketch of the link between thermocline change and how it is reflected in our biomarker data. Shades and gradients indicate the export depth of respective biomarkers; green shade=photic zone, blue shade=NO3- rich zone, red line=temperature profile; solid arrows indicate production, dashed arrows indicate to which depth the proxy is calibrated. A thermocline shoaling has the largest effect on TEX86, since the GDGTs on which this index is based are produced and exported predominantly from the depth close to the thermocline. Note that TEX86 records that are translated into SSTs will reflect the same trend, as they are presumed to have a constant offset with the subsurface temperatures they actually represent.

Figure redacted

[redacted]

Line 164-165: It is hard to reconcile the stated "weaken AMOC upper limb" and "increased overturning" at the same time because the upper limb and the lower limb should change in-phase as the lower limb is the return flow of the AMOC. The core of the AMOC 4upper cell locates at ~750m. If the AMOC at MIS M2 is with similar geometry as in mPWP, it seems the AMOC upper cell is weaker from Figure 4b. The AMOC at MIS M2 should be provided in addition to the anomaly plot. The strength of AMOC is usually defined as the maximum overturning streamfunction in the North Atlantic at mid-depth. The specific values of AMOC strength at mPVP and MIS M2 should be provided.

Correction: We now provide a separate MIS M2 AMOC panel and the specific AMOC streamfunction values as suggested by the reviewer. We also rephrase this part to make it clearer. Please refer to Fig. 4 and lines 190-196.

Line 180-181: Why the decrease of the temperature gradient between 40N and 53N infers a decrease suppler of NAC? NAC transports heat to the high latitude, therefore stronger NAC should lead to weaker SST gradient.

Reply: We thank the reviewer for noticing this; this was a mistake. There was an increase in the SST gradient during 3400Ka-MIS M2. We now clarify this. Regardless, the general cooling trend along the NAC pathway is the main evidence for a reduced NAC influence.

Correction: Please refer to lines 208-209.

Line 182-183: The salinity change in the Caribbean Sea could also be affected by AAIW.

Reply: We agree with the reviewer, but the mechanisms driving Caribbean Sea salinity are beyond the scope of our study. Here, we mainly want to emphasize that Caribbean Sea salinity is not coupled to AL.

Line 204: It is more appropriate to identify the NADW formation region from the winter mixed layer depth.

Correction: We have added a maximum winter mixed layer depth figure for the mPWP and MIS M2 as Extended data fig. 9.

Line 207: What is the dynamical mechanism between thermocline shoaling and NADW intensification?

Reply: We have deliberately refrained from proposing a causal link and chosen to remain speculative. Thermocline shoaling and NADW intensification likely act/react together under a cooling climate. Nevertheless, there has been a mechanism previously proposed: In a first-baroclinic framework, stronger deepwater formation acts as a sink of upper-layer mass, leading to a thinning of the upper ocean and an upward displacement of the main thermocline. This adjustment propagates across basins via Kelvin and Rossby waves (Huang et al., 2000). This very general theorical process based on a relatively simple model has been observed to occur in some paleo proxy studies (e.g., Lopes dos Santos et al., 2010; Steph et al., 2010), and now in our work. Thus, enhanced overturning can generate basin-scale thermocline shoaling independent of purely local wind-driven upwelling (Chiang et al., 2008), offering a dynamical explanation for the coherent subsurface signal observed at multiple Atlantic sites.

Correction: We have briefly summarised the content above in lines 172-173 and provide corresponding references.

Reference:

Toggweiler, J. R., & Samuels, B. (1995). Effect of drake passage on the global thermohaline circulation. Deep-Sea Research Part I, 42(4), 477–500. https://doi.org/10.1016/0967-0637(95)00012-U

References

Bijl, P. K., Śliwińska, K. K., Duncan, B., Huguet, A., Naeher, S., Rattanasriampaipong, R., Sosa-Montes de Oca, C., Auderset, A., Berke, M. A., Kim, B. S., Davtian, N., Dunkley Jones, T., Eefting, D. D., Elling, F. J., Fenies, P., Inglis, G. N., O'Connor, L., Pancost, R. D., Peterse, F., Rice, A., Sluijs, A., Varma, D., Xiao, W., and Zhang, Y. G.: Reviews and syntheses: Best practices for the application of marine GDGTs as proxy for paleotemperatures: sampling, processing, analyses, interpretation, and archiving protocols, Biogeosciences, 22, 6465–6508, https://doi.org/10.5194/bg-22-6465-2025, 2025.

Caley, T., Giraudeau, J., Malaizé, B., Rossignol, L., and Pierre, C.: Agulhas leakage as a key process in the modes of Quaternary climate changes, Proceedings of the National Academy of Sciences, 109, 6835–6839, https://doi.org/10.1073/pnas.1115545109, 2012.

Chiang, J. C. H., Cheng, W., and Bitz, C. M.: Fast teleconnections to the tropical Atlantic sector from Atlantic thermohaline adjustment, Geophysical Research Letters, 35, https://doi.org/10.1029/2008GL033292, 2008.

Huang, R. X., Cane, M. A., Naik, N., and Goodman, P.: Global adjustment of the thermocline in response to deepwater formation, Geophysical Research Letters, 27, 759–762, https://doi.org/10.1029/1999GL002365, 2000.

Lopes dos Santos, R. A., Prange, M., Castañeda, I. S., Schefuß, E., Mulitza, S., Schulz, M., Niedermeyer, E. M., Sinninghe Damsté, J. S., and Schouten, S.: Glacial–interglacial variability in Atlantic meridional overturning circulation and

thermocline adjustments in the tropical North Atlantic, Earth and Planetary Science Letters, 300, 407–414, https://doi.org/10.1016/j.epsl.2010.10.030, 2010.
Lu, L., Zheng, X., Weber, M. E., Peck, V., Reilly, B. T., Chen, Z., Yan, W., Chen, T., Yan, H., Gong, X., Wu, S., Zheng, L., Wan, S., Du, Y., Tauxe, L., Yang, Q., Brachfeld, S., Williams, T., Martos, Y. M., Du, Z., Garcia, M., Pérez, L. F., Yang, H., Huang, B., Warnock, J., and Kao, S.-J.: Extremely poleward shift of Antarctic Circumpolar Current by eccentricity during the Last Interglacial, Nat Commun, 16, 8869, https://doi.org/10.1038/s41467-025-63933-x, 2025.
Petrick, B., McClymont, E. L., Felder, S., Rueda, G., Leng, M. J., and Rosell-Melé, A.: Late Pliocene upwelling in the Southern Benguela region, Palaeogeography, Palaeoclimatology, Palaeoecology, 429, 62–71, https://doi.org/10.1016/j.palaeo.2015.03.042, 2015.
Petrick, B., McClymont, E. L., Littler, K., Rosell-Melé, A., Clarkson, M. O., Maslin, M., Röhl, U., Shevenell, A. E., and Pancost, R. D.: Oceanographic and climatic evolution of the southeastern subtropical Atlantic over the last 3.5 Ma, Earth and Planetary Science Letters, 492, 12–21, https://doi.org/10.1016/j.epsl.2018.03.054, 2018.
Schouten, S., Hopmans, E. C., Rosell-Melé, A., Pearson, A., Adam, P., Bauersachs, T., Bard, E., Bernasconi, S. M., Bianchi, T. S., Brocks, J. J., Carlson, L. T., Castañeda, I. S., Derenne, S., Selver, A. D., Dutta, K., Eglinton, T., Fosse, C., Galy, V., Grice, K., Hinrichs, K.-U., Huang, Y., Huguet, A., Huguet, C., Hurley, S., Ingalls, A., Jia, G., Keely, B., Knappy, C., Kondo, M., Krishnan, S., Lincoln, S., Lipp, J., Mangelsdorf, K., Martínez-García, A., Ménot, G., Mets, A., Mollenhauer, G., Ohkouchi, N., Ossebaar, J., Pagani, M., Pancost, R. D., Pearson, E. J., Peterse, F., Reichart, G.-J., Schaeffer, P., Schmitt, G., Schwark, L., Shah, S. R., Smith, R. W., Smittenberg, R. H., Summons, R. E., Takano, Y., Talbot, H. M., Taylor, K. W. R., Tarozo, R., Uchida, M., van Dongen, B. E., Van Mooy, B. A. S., Wang, J., Warren, C., Weijers, J. W. H., Werne, J. P., Woltering, M., Xie, S., Yamamoto, M., Yang, H., Zhang, C. L., Zhang, Y., Zhao, M., and Damsté, J. S. S.: An interlaboratory study of TEX86 and BIT analysis of sediments, extracts, and standard mixtures, Geochemistry, Geophysics, Geosystems, 14, 5263–5285, https://doi.org/10.1002/2013GC004904, 2013.
Schulzki, T., Schwarzkopf, F. U., and Biastoch, A.: Atlantic Meridional Overturning Response to Increased Southern Ocean Wind Stress in a Climate Model with an Eddy-Rich Ocean, Journal of Climate, 37, 5769–5792, https://doi.org/10.1175/JCLI-D-23-0727.1, 2024.
van Sebille, E., Biastoch, A., van Leeuwen, P. J., and de Ruijter, W. P. M.: A weaker Agulhas Current leads to more Agulhas leakage, Geophysical Research Letters, 36, https://doi.org/10.1029/2008GL036614, 2009.
Starr, A., Hall, I. R., Barker, S., Nederbragt, A., Owen, L., and Hemming, S. R.: Shifting Antarctic Circumpolar Current south of Africa over the past 1.9 million years, Science Advances, 11, eadp1692, https://doi.org/10.1126/sciadv.adp1692, 2025.
Steph, S., Tiedemann, R., Prange, M., Groeneveld, J., Schulz, M., Timmermann, A., Nürnberg, D., Rühlemann, C., Saukel, C., and Haug, G. H.: Early Pliocene increase in thermohaline overturning: A precondition for the development of the modern equatorial

Pacific cold tongue: PLIOCENE EAST PACIFIC COLD TONGUE STATE, Paleoceanography, 25, https://doi.org/10.1029/2008PA001645, 2010.

Toggweiler, J. R. and Samuels, B.: Effect of drake passage on the global thermohaline circulation, Deep Sea Research Part I: Oceanographic Research Papers, 42, 477–500, https://doi.org/10.1016/0967-0637(95)00012-U, 1995.

Van der Weijst, C. M. H., van der Laan, K. J., Peterse, F., Reichart, G.-J., Sangiorgi, F., Schouten, S., Veenstra, T. J. T., and Sluijs, A.: A 15-million-year surface- and subsurface-integrated TEX_{86} temperature record from the eastern equatorial Atlantic, Climate of the Past, 18, 1947–1962, https://doi.org/10.5194/cp-18-1947-2022, 2022a.

Van der Weijst, C. M. H., Winkelhorst, J., de Nooijer, W., von der Heydt, A., Reichart, G.-J., Sangiorgi, F., and Sluijs, A.: Pliocene evolution of the tropical Atlantic thermocline depth, Climate of the Past, 18, 961–973, https://doi.org/10.5194/cp-18-961-2022, 2022b.

Wu, S., Mazaud, A., Michel, E., Erb, M. P., Stocker, T. F., Amsler, H. E., Le Tallec-Carado, P., Lamy, F., and Jaccard, S. L.: Zonally asymmetric changes in the Antarctic Circumpolar Current strength over the past million years, Nat. Geosci., 1–8, https://doi.org/10.1038/s41561-025-01901-2, 2026.

Referee comments:

Reviewer #1 (Remarks to the Author):

I was asked to re-review the article "Agulhas Leakage disconnected to Atlantic Meridional Overturning in the Late Pliocene". I would like to start by thanking the authors for their response to the reviewers and for their very organized approach to presenting the corrections. I also appreciate that, in the rare cases when they disagree with my suggestions, they explain their reasoning thoughtfully. I find the organization really brings out the major point, which I think makes this article hugely significant: the connections between the AL, STF, and AMOC are much more complex than initial studies have suggested. I recommend strengthening the final statement and making it clear that this may not hold for the 100 ka world, as several studies have suggested. However, I will leave that up to the authors. I have one note:

Age Models: Can you add a table with tie points for the new adjusted age model or provide a link to where such a table can be found? From experience, this will help future researchers who work on this core.

I think you can send this directly to the editor during the proofs stage.

Thanks again for your hard work and congratulations on a significant study.

Dr. Benjamin Petrick

Reply: We thank Dr. Petrick for the positive comments and constructive suggestions. We are glad that our revised interpretation is now better reconciled with previously published data.

Corrections: Regarding the comment that "this may not hold for the 100ka world", we have already emphasized the limitation in the final sentence as "at least in the Late Pliocene". We would therefore prefer not to elaborate further on this point in order to avoid introducing additional uncertainties beyond the scope of the present study.

We have added a table of the U1475 age model tie points in the supplementary information.

Reviewer #2 (Remarks to the Author):

I thank the authors for carefully addressing my previous comments, particularly their inclusion of wind forcing effects on Agulhas Leakage and the added discussion on the driving mechanisms of the AMOC. These revisions have improved the clarity and completeness of the manuscript. I have no further questions or concerns.

Reply: We thank the reviewer for the constructive comments provided in the previous review round and for the positive evaluation of our revisions. We are pleased that the revised manuscript has addressed the reviewer's concerns.
